# Do irrigating solutions influence the cyclic fatigue resistance of heat-treated NiTi instruments? an in vitro study

**DOI:** 10.1007/s44445-025-00093-0

**Published:** 2026-01-23

**Authors:** Christoph Matthias Schoppmeier, Malin Janson, Li Sun, Gustav Leo Classen, Anna Greta Barbe

**Affiliations:** 1 Polyclinic for Operative Dentistry and Periodontology, University of Cologne, Faculty of Medicine and University Hospital Cologne, Kerpener Str. 32, 50931 Cologne, Germany; 2 Department of Prosthetic Dentistry, University of Cologne, Faculty of Medicine and University Hospital Cologne, Cologne, Germany

**Keywords:** Nickel-Titanium instruments, Cyclic fatigue resistance, Endodontic irrigants, Surface corrosion, Sodium hypochlorite, Ethylenediaminetetraacetic acid (EDTA)

## Abstract

**Purpose:**

Nickel–titanium (NiTi) instruments have advanced root canal preparation through enhanced precision. Thermal pretreatment improves both flexibility and cyclic fatigue resistance (CFR). While irrigating solutions are essential for decontamination, they may also affect the properties of heat-treated NiTi instruments. This study aimed to evaluate the impact of different irrigating solutions on the cyclic fatigue resistance of heat-treated NiTi files.

**Methods:**

Four heat-treated reciprocating NiTi-files were analyzed: EdgeOne R-Utopia, Reciproc Blue, Procodile Q and CC One Blue. Files were immersed (5 min; 37 °C and 60 °C) in 5.25% sodium hypochlorite (NaOCl), 17% ethylenediaminetetraacetic acid (EDTA), 96% ethanol, NaOCl + EDTA, distilled water, or left in a no-immersion control group. CFR was measured in an artificial root canal (60° curvature, 5 mm radius), and fragment length (FL) was documented. The surface features of the fragments were assessed through scanning electron microscopy.

**Results:**

File system, irrigating solution, and temperature significantly influenced CFR (p < 0.001). Across all immersion conditions, the CFR reached its highest value with Procodile Q (37 °C distilled water) and its lowest with EdgeOne R-Utopia (60 °C NaOCl + EDTA). EDTA reduced CFR across all files, particularly at elevated temperatures and when combined with NaOCl. Microscopy revealed micropitting and roughened surfaces, particularly on CC One Blue (60 °C NaOCl), as well as material degradation and heterogeneous surfaces with NaOCl + EDTA.

**Conclusions:**

Heat-treated NiTi instruments are influenced in their mechanical and metallurgical behavior by the chemical and thermal impact of irrigating solutions. Heated EDTA and NaOCl + EDTA were detrimental, while Procodile Q demonstrated the highest CFR. Within the limitations of this study, exposure to irrigating solutions, particularly heated EDTA and NaOCl + EDTA, reduced the cyclic fatigue resistance of heat-treated NiTi files. Clinically, prudent selection and temperature control of irrigants may help preserve instrument performance and reduce the risk of file fracture during root canal preparation.

**Supplementary Information:**

The online version contains supplementary material available at 10.1007/s44445-025-00093-0.

## Introduction

Because of their outstanding flexibility, shape memory alloy characteristics, and high cyclic fatigue resistance, nickel-titanium (NiTi) files have become crucial in endodontic therapy (Walia et al. 1988; Adigüzel and Turgay 2017). By enabling efficient, conservative, and precise canal preparation, these properties decrease the risk of iatrogenic complications and play an important role in clinical prognosis (Thu et al. 2020)). However, cyclic fatigue caused by repeated mechanical loading during root canal preparation remains one of the main causes of instrument fractures, complicating clinical procedures (Sattapan et al. 2000).

Thermal treatment of NiTi files has improved flexibility and cyclic fatigue resistance (CFR), thereby reducing fracture risk (Peters et al. 2012). Among reciprocating, thermally treated NiTi file systems, four representative examples are frequently used in clinical practice: Procodile Q, EdgeOne R-Utopia, CC One Blue, and Reciproc Blue. Procodile Q (Komet Dental, Lemgo, Germany) and EdgeOne R-Utopia (Edge Endo, Albuquerque, USA) undergo low-temperature heat treatment (≈400 °C), producing a brown-gold oxide surface layer. Reciproc Blue (VDW GmbH, Munich, Germany) is subjected to high-temperature treatment (450–500 °C), which induces martensitic transformation and creates a characteristic blue oxide layer, thereby enhancing flexibility and fracture resistance (De-Deus et al. 2017). CC One Blue (Bondent, San Clemente, USA) is manufactured from M-Wire alloy with an additional thermal nano-coating for surface refinement. Notably, Procodile Q is unique in being machined after heat treatment, a modification that may influence its surface integrity and mechanical performance (Bürklein et al. 2024; Mena-Álvarez et al. 2022; Schoppmeier et al. 2025).

In addition to mechanical stresses, thermal and chemical factors, particularly those associated with irrigating solutions, significantly affect the longevity and cyclic fatigue resistance (CFR) of NiTi instruments by altering their metallurgical and physical properties (Ríos-Osorio et al. 2024). Irrigation is essential in chemomechanical root canal preparation, as it removes smear layers and necrotic tissue, disrupts biofilms, and disinfects dentin surfaces. Among irrigants, sodium hypochlorite (NaOCl) remains the gold standard due to its antimicrobial activity and tissue-dissolving ability. Its effectiveness can be enhanced by increasing concentration, volume, agitation, or thermal activation (Zehnder 2006). Adjunctive techniques such as ultrasonic or sonic activation as well as laser-assisted irrigation have also been shown to improve penetration and efficacy. Thermal activation of NaOC through preheating increases its antimicrobial effect and dentinal tubule penetration. However, these methods must balance enhanced disinfection with concerns regarding solution stability, handling safety, and effects on dentin structure (Yared and Al Asmar Ramli 2020; Damade et al. 2020). The active chlorine ions in NaOCl not only disrupt organic tissues but also promote corrosion of NiTi alloys (Reis-Prado et al. 2023). Corrosion patterns such as micropitting or selective nickel dissolution generate surface defects that act as stress concentrators and thereby reduce CFR (Keles et al. 2019).

Ethylenediaminetetraacetic acid (EDTA) is often used in combination with NaOCl to remove the inorganic fraction of the smear layer through calcium chelation (Carvalho et al. 2008). This improves irrigant penetration but also introduces risks. Sequential or simultaneous use with NaOCl may produce precipitates and promote corrosion, further compromising instrument integrity (Tartari et al. 2017). While the effects of NaOCl heating are well studied, the influence of thermally activated EDTA on NiTi corrosion and CFR remains unclear.

Ethanol is sometimes applied as a final rinse because its volatility accelerates canal drying and optimizes obturation conditions. Yet, its impact on corrosion and NiTi structural stability is insufficiently investigated (Mampilly et al. 2020). Thus, while irrigants enhance disinfection, their combined thermal, chemical, and mechanical interactions with NiTi alloys can compromise surface integrity and ultimately reduce CFR. Previous studies have evaluated the impact of heat treatment and irrigation on NiTi instruments in various reciprocating and rotary systems (Keles et al. 2019; Scott et al. 2019; Klymus et al. 2019). While improvements in flexibility and CFR have been reported, findings regarding the combined chemical and thermal effects of irrigating solutions are inconsistent. Importantly, little is known about the role of ethanol and the influence of irrigation protocols on differently heat-treated file systems. This gap in the literature highlights the need for systematic investigation.

The purpose of this study was to investigate the impact of different irrigating solutions (NaOCl, EDTA, ethanol) and conventional irrigation protocols on NiTi instruments subjected to high- and low-temperature heat treatments. Our focus was to evaluate how the chemical and thermal characteristics of the irrigating solutions would affect metallurgical properties, mechanical durability and corrosion susceptibility of the NiTi-files. Accordingly, our in vitro study tested the following null hypothesis: the chemical composition and thermal activation of irrigating solutions do not induce significant metallurgical alterations that impact the CFR of high- or low-temperature heat-treated NiTi instruments.

## Materials and methods

**Fig. 1:**
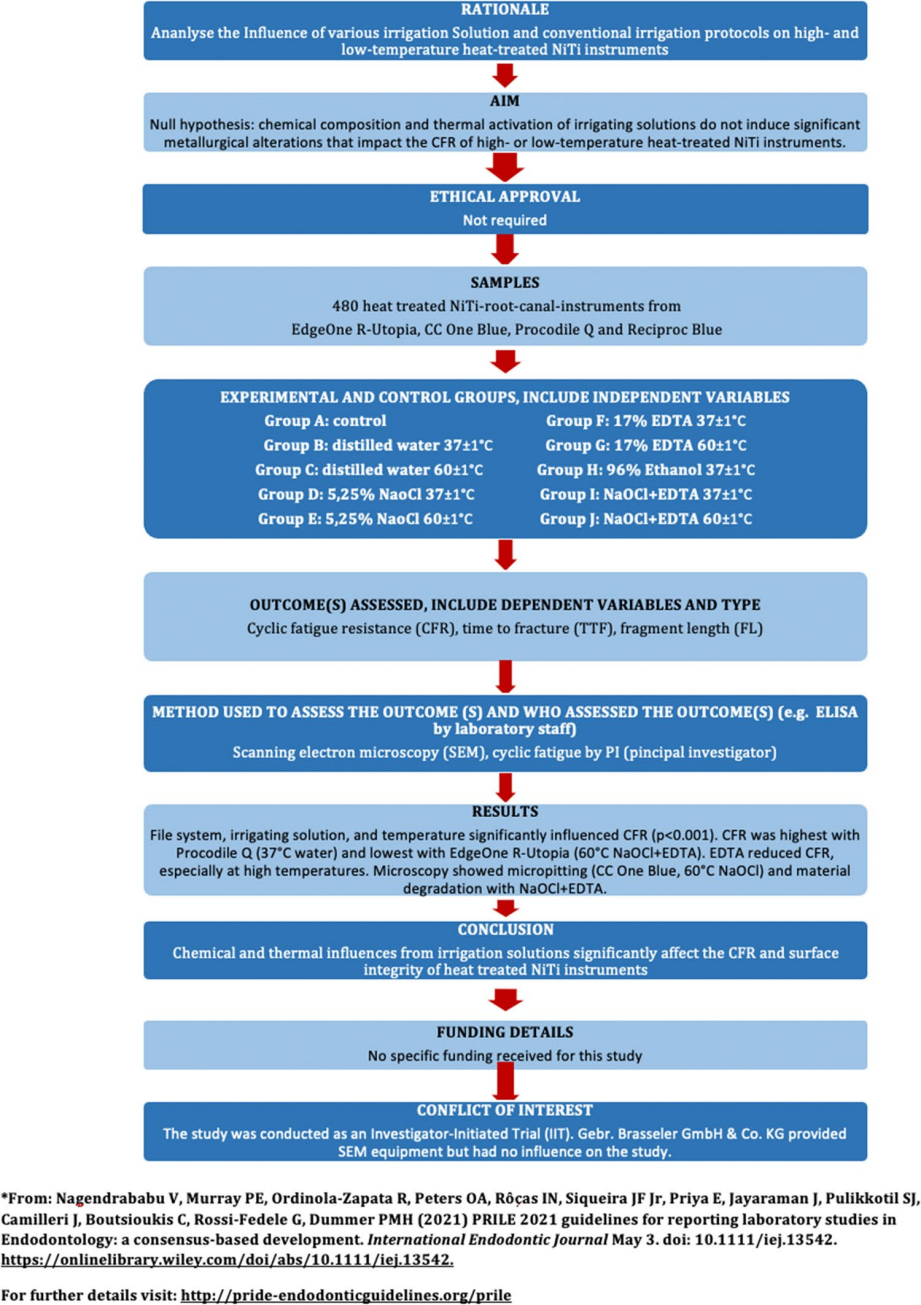
PRILE Flowchart

The manuscript of this laboratory study has been written according to Preferred Reporting Items for Laboratory studies in Endodontology (PRILE) 2021 guidelines (Nagendrababu et al. 2021, 10.1111/iej.13542). All experiments were conducted at the Department of Operative Dentistry and Periodontology, University of Cologne, Germany (Fig. 1).

Prior to the in vitro analysis, the files were evaluated under 25 × magnification with a VHX-5000 microscope (Keyence Corp., Osaka, Japan) to detect defects or deformations, and no abnormalities were found that necessitated exclusion.

### Sample size

Using data from a study with ten samples (Keles et al. 2019), the sample size was recalculated with the help of G*Power v3.1 (Heinrich Heine University Düsseldorf, Düsseldorf, Germany), based on the effect sizes observed for CFR (mean ± standard deviation of the number of cycles to fracture) in different reciprocating file systems. It was determined that a minimum of 12 samples per immersion group was required to detect significant differences at a 5% significance level and 80% statistical power. Due to the limited information on the mechanical characteristics of CC One Blue, Procodile Q, and EdgeOne R-Utopia, the sample size was conservatively increased to 15 specimens per group to enhance the reliability and robustness of the results.

### Immersion

Under standardized conditions, the working segments of the files (16 mm) were submerged for 5 min in the following irrigating solutions (Cai et al. 2017): A) control: no immersion; B) distilled water (37 ± 1 °C); C) distilled water (60 ± 1 °C); D) 5.25% NaOCl (37 ± 1 °C); E) 5.25% NaOCl (60 ± 1 °C); F) 17% EDTA (37 ± 1 °C); G) 17% EDTA (60 ± 1 °C); H) 96% ethanol (37 ± 1 °C); I) 5.25% NaOCl + 17% EDTA (37 ± 1 °C, alternating every minute, starting with NaOCl); J) 5.25% NaOCl + 17% EDTA (60 ± 1 °C, alternating every minute, starting with NaOCl). The immersion time was standardized to 5 min for all groups, in accordance with previous in-vitro protocols investigating irrigant–NiTi interactions (Carvalho et al. 2008).

### Cyclic fatigue resistance

**Fig. 2:**
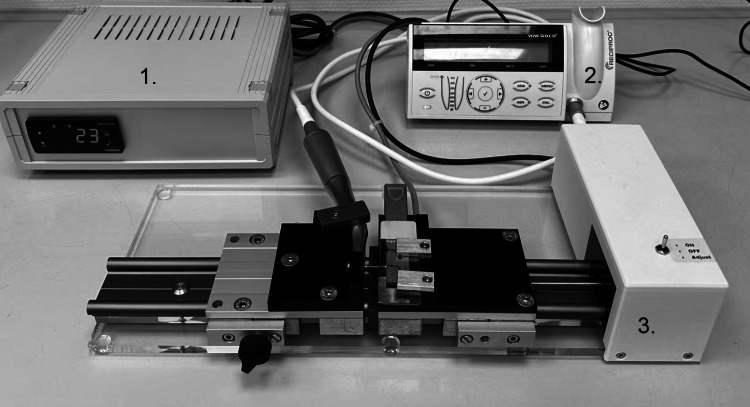
Device used for cyclic fatigue resistance (CFR) testing. (1) A temperature regulator linked to heating plates (Tru Components, 7.5 Ohm, 20 W, Tru Components, USA) maintained the target temperature (room temperature, 22 °C). (2) A electric motor with torque controll (VDW Gold, VDW, Munich, Germany) was used to provide precise control throughout the testing process. (3) The CFR testing device included a handpiece and an artificial root canal to conduct the experimental procedures

CFR testing was performed using a custom-built apparatus (Fig. 2), adapted from previously established protocols (Keles et al. 2019; Erik and Özyürek 2019; Pedullà et al. 2014; Uslu et al. 2018). The system, constructed with durable metal components, made it possible to position the files accurately inside an artificial root canal. The canal simulated a single curvature of 60° with a 5 mm radius, with the curvature center located 6 mm from the file tip. The tests were performed at room temperature (22 °C) under static conditions (Reis-Prado et al. 2023). All files were tested in the “RECIPROC ALL” mode at 300 rpm, using a VDW Gold motor (VDW, Munich, Germany), as per the manufacturers’ instructions. A lubricant (WD 40, WD Company, Milton Keynes, UK) was applied to lower friction. It was applied directly into the artificial canal of the cyclic fatigue testing device. The files were subjected to stress until the fractured. A digital chronometer (0.01 s accuracy) was used to measure time to fracture (TTF), and measurements were validated through video documentation. The fractured files’ fragment lengths (FL) were assessed with a digital caliper.

### Scanning electron microscopy

To investigate surface structures and material modifications, high-resolution SEM images were acquired from two fragments of each immersion group (A, E, G, J) using an SEM system (EVO MA 10, Carl Zeiss, Oberkochen, Germany). Before SEM imaging, the fragments were rinsed with distilled water, cleaned ultrasonically in 96% ethanol, and prepared for analysis.

### Statistical analysis

A three-way ANOVA was conducted in R 4.3.0 (R Foundation for Statistical Computing, Vienna, Austria) to analyze both TTF and FL. The effects of file type, immersion method and their interaction on TTF and FL were assessed. The homoscedasticity of the residuals was examined using Levene’s test, and normality was assessed with the Shapiro–Wilk test. An alpha level of 5% was set for statistical significance. Pairwise differences between groups (defined by file type and immersion method) were analyzed using Tukey-HSD post hoc tests. Potential interaction effects among independent variables were also considered to differentiate their influence on TTF. The results are expressed as mean values with their corresponding standard deviations.

## Results

### TTF analysis

The data of the in vitro mechanical tests for TTF (means ± standard deviations) and FL are shown in Table 1. The results of the Shapiro–Wilk test indicated that the data did not deviate significantly from normal distribution (p > 0.05). The Analysis showed significant variations between the tested file systems, immersion conditions, and their interaction (p < 0.001). Post-hoc testing revealed that Procodile Q exhibited significantly higher TTF values than EdgeOne R-Utopia across all immersion conditions (p < 0.001). Compared with CC One Blue, Procodile Q was also significantly superior in most conditions (e.g., Ethanol 37 °C p < 0.001; EDTA 37 °C p < 0.001; EDTA 60 °C p < 0.001; NaOCl 37 °C p < 0.001; NaOCl 60 °C p < 0.001; NaOCl + EDTA 37/60 °C p < 0.001), while differences were not significant in Control (p = 0.097) and H₂O 37 °C (p = 0.628). Detailed pairwise results are presented in Supplementary Table 1.

**Table 1:** Descriptive Statistics of cyclic fatigue testing with TTF (mean and SD) of each NiTi file system tested

	Procodile Q		EdgeOne R-Utopia		Reciproc blue		CC One Blue	
Immersion	TTF	FL	TTF	FL	TTF	FL	TTF	FL
Control	297.1 $\pm$ 29.9^Aa^	6.23 $\pm$ 1.11	151.4 $\pm$ 29.7^Ac^	7.43 $\pm$ 0.86	230.4 $\pm$ 32.4^Ab^	6.80 $\pm$ 0.87	234 $\pm$ 31.9^Aab^	6.99 $\pm$ 1.51
Distilled H_2_O 37 °C	324.5 $\pm$ 64.8^Aa^	6.58 $\pm$ 0.99	142.7 $\pm$ 27.4^Ab^	6.98 $\pm$ 0.68	242.8 $\pm$ 26.4^Aa^	6.49 $\pm$ 2.04	252.3 $\pm$ 22.7^Aa^	7.12 $\pm$ 0.73
Distilled H_2_O 60 °C	310.6 $\pm$ 22.2^Aa^	7.18 $\pm$ 1.43	140.1 $\pm$ 26.8^Ab^	6.48 $\pm$ 1.23	228.1 $\pm$ 25.6^Aa^	5.97 $\pm$ 2.09	230.5 $\pm$ 29.9^Aa^	6.63 $\pm$ 0.69
5,25% NaOCL 37 °C	289.2 $\pm$ 46^Aa^	6.88 $\pm$ 1.05	117.2 $\pm$ 17.3^Bc^	7.13 $\pm$ 0.88	225.3 $\pm$ 32.8^Ab^	7.77 $\pm$ 0.56	200.1 $\pm$ 39.2^Bab^	6.41 $\pm$ 1.21
5,25% NaOCL 60 °C	263.0 $\pm$ 33.1^Aa^	6.73 $\pm$ 1.60	109.7 $\pm$ 18.3^Bc^	7.24 $\pm$ 1.25	216.8 $\pm$ 21.8^Bb^	6.00 $\pm$ 1.69	110.7 $\pm$ 26.2^Cab^	6.88 $\pm$ 0.42
17% EDTA 37 °C	260.9 $\pm$ 48.8^Aa^	6.63 $\pm$ 1.35	99.6 $\pm$ 30.5^Bc^	6.78 $\pm$ 1.16	200.9 $\pm$ 21.6^Bb^	6.69 $\pm$ 1.52	197.2 $\pm$ 33^Bab^	5.86 $\pm$ 1.68
17% EDTA 60 °C	221.2 $\pm$ 43.3^Ba^	6.55 $\pm$ 1.29	85.3 $\pm$ 11.2^Cc^	7.44 $\pm$ 1.18	186.3 $\pm$ 24.8^Bab^	6.17 $\pm$ 1.73	177 $\pm$ 25.6^Bb^	6.19 $\pm$ 1.68
96% Ethanol 37 °C	301.1 $\pm$ 12.66^Aa^	6.37 $\pm$ 0.88	143.8 $\pm$ 22.8^Ac^	6.64 $\pm$ 0.64	222.2 $\pm$ 28.3^Ab^	6.73 $\pm$ 1.52	196.5 $\pm$ 30.9^Ab^	6.83 $\pm$ 0.86
5,25% NaOCL + 17% EDTA 37 °C	207.7 $\pm$ 16.7^Ca^	6.20 $\pm$ 1.64	110.4 $\pm$ 30.8^Bc^	5.91 $\pm$ 1.19	191.2 $\pm$ 26.8^Cb^	6.65 $\pm$ 1.54	140.7 $\pm$ 36.7^Cb^	6.06 $\pm$ 1.24
5,25% NaOCL + 17% EDTA 60 °C	170.3 $\pm$ 32.7^Ca^	6.81 $\pm$ 1.43	68.5 $\pm$ 33.95^Dc^	5.26 $\pm$ 0.98	158 $\pm$ 18.41^Cb^	5.91 $\pm$ 1.71	95.9 $\pm$ 27.61^Cb^	6.26 $\pm$ 1.38

EDTA, Ethylenediaminetetraacetic acid; FL, File Length; NaOCL, Sodium hypochlorite; SD, Standard deviation; TTF, Time to fracutre; Different lowercase superscript letters within a row indicate significant differences between file systems under the same condition, whereas different uppercase superscript letters within a column indicate significant differences between conditions within the same file system (Tukey’s HSD, α = 0.05)

### CFR analysis

Procodile Q demonstrated the highest CFR across all immersion conditions (maximum TTF: 324.5 ± 64.8 s in 37 °C distilled water). Conversely, EdgeOne R-Utopia showed the lowest fatigue resistance (minimum TTF: 68.5 ± 33.95 s in 60 °C NaOCl + EDTA). Compared with EdgeOne R-Utopia, Procodile Q had significantly higher CFR values under every medium/temperature (p < 0.001).

EDTA markedly reduced TTF, particularly at elevated temperatures. For example, Procodile Q decreased from 324.5 ± 64.8 s in 37 °C distilled water to 221.2 ± 43.3 s in 60 °C EDTA (p < 0.001), and EdgeOne dropped significantly from Control to 60 °C EDTA (p < 0.001). Not all pairings were significant, however (e.g., CC One H₂O 60 °C vs EDTA 60 °C: p = 0.231).

**Fig. 3:**
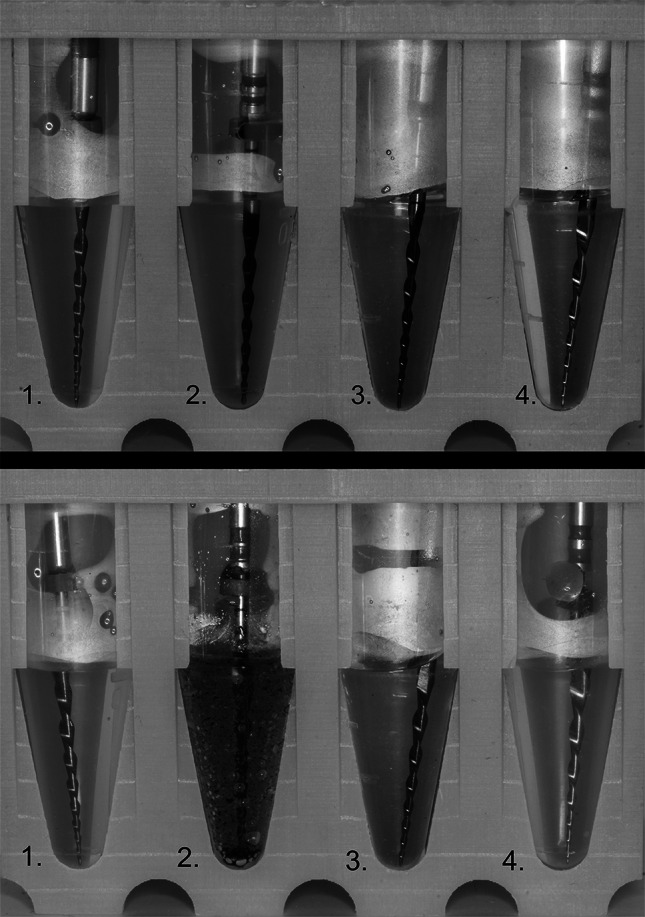
Immersion of the tested file systems: (1) Reciproc Blue, (2) CC One Blue, (3) EdgeOne R-Utopia, (4) Procodile Q. The top image shows immersion in 60 °C distilled water, while the bottom image depicts immersion in 60 °C sodium hypochlorite (NaOCl). Noticeable dissolution, turbidity and chemical reactions are evident in (2) CC One Blue, indicating an interaction between NaOCl and the nano-coating

NaOCl immersion caused minor to moderate TTF reductions, with CC One Blue showing heightened sensitivity. Its TTF decreased from 200.1 ± 39.2 s (37 °C NaOCl) to 110.7 ± 26.2 s (60 °C NaOCl; p < 0.001). In contrast, several comparisons in other systems were not significant (e.g., EdgeOne Control vs NaOCl 60 °C p = 0.656; Reciproc Blue Control vs NaOCl 60 °C p = 1.0; Procodile Q Control vs NaOCl 60 °C p = 0.965). Immersion in 60 °C NaOCl produced a distinct blue-black discoloration in CC One Blue (Fig. 3).

The combination NaOCl + EDTA had the most severe impact, yielding the lowest TTF values, especially at 60 °C. Procodile Q dropped to 170.3 ± 32.7 s, Reciproc Blue to 158.0 ± 18.4 s, CC One Blue to 95.9 ± 27.6 s, and EdgeOne R-Utopia to 68.5 ± 33.95 s. For all systems, H₂O 37 °C vs NaOCl + EDTA 60 °C was highly significant (p < 0.001).

### Fragment length and surface analysis

Significant variations among the file systems (p = 0.004) and immersion conditions (p = 0.004) were observed in the FL analysis. EdgeOne R-Utopia and CC One Blue showed longer FL, particularly at higher temperatures (e.g. 60 °C) and in EDTA solutions (Table 1).

**Fig. 4:**
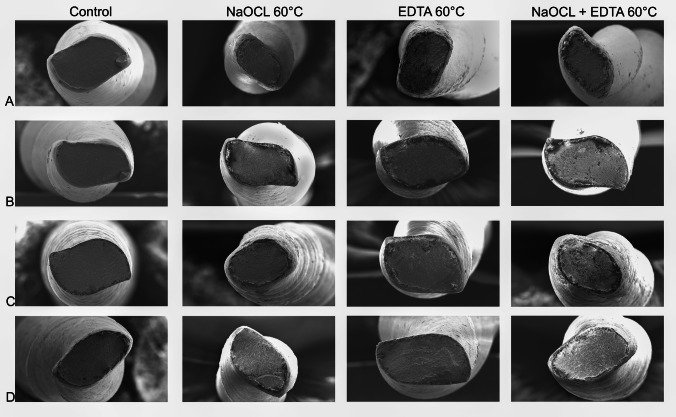
Scanning electron microscopy (SEM) images of fractured surfaces from cyclic fatigue testing. SEM micrographs show the fractured surfaces of **A**) Procodile Q, **B**) EdgeOne R-Utopia, **C**) Reciproc Blue, and **D**) CC One Blue instruments after cyclic fatigue testing under various immersion conditions: control, 60 °C sodium hypochlorite (NaOCl), 60 °C ethylenediaminetetraacetic acid (EDTA), and 60 °C NaOCl + EDTA. Fatigue striations typical of cyclic fatigue are visible on the fractured surfaces, along with surface alterations caused by chemical interactions during immersion

The fracture cross-sections analyzed by SEM (Fig. 4) exhibited characteristic signs of cyclic fatigue, including the origin of cracks, distinct fatigue zones, and an overload area. Notable findings included micropitting on CC One Blue after immersion in 60 °C NaOCl, suggesting increased surface sensitivity. EDTA treatments caused rougher, more uneven surface structures across all systems, with the most pronounced changes observed in the NaOCl + EDTA combination, resulting in severe material degradation and heterogeneous surfaces.

## Discussion

Our results clearly demonstrate that both the chemical composition and thermal activation of irrigating solutions significantly affected the CFR of NiTi instruments, thus refuting our null hypothesis. Our key findings included a significant reduction in CFR among NiTi files exposed to heat and EDTA, particularly the sequential use of NaOCl and EDTA, which further accelerated material degradation (ethanol had no significant effects on the CFR). No significant differences in CFR were detected between high- and low-temperature heat-treated NiTi files, indicating that the type of thermal pretreatment alone does not confer superior resistance under chemical exposure. This manufacturing approach may contribute to the observed resistance of Procodile Q to the tested irrigating solutions, potentially offering some degree of protection and reducing corrosion susceptibility (Keles et al. 2019; Erik and Özyürek 2019; Huang et al. 2017; Elnaghy and Elsaka 2017).

Immersion in NaOCl resulted in moderate reductions in CFR, aligning with previous studies that have reported NaOCl-induced CFR decreases in NiTi instruments across various concentrations (Reis-Prado et al. 2023). Keles et al. highlighted that the temperature of NaOCl significantly affected the CFR of heat-treated Reciproc files (Keles et al. 2019). Microstructural changes such as micropitting and selective nickel dissolution caused by NaOCl lead to stress concentrations and crack formation, ultimately weakening the fatigue resistance of the instruments (Keles et al. 2019). At elevated temperatures, NaOCl further impacts the crystallographic arrangement, triggering reverse martensitic phase transformation and reducing flexibility (Grande et al. 2017). According to Ametrano et al., a 5-min exposure to 5.25% NaOCl led to surface pitting and crack formation on NiTi instruments, negatively affecting their fracture resistance and overall integrity. (Ametrano et al. 2011). Similar observations in this study were evident for EdgeOne R-Utopia and CC One Blue. Notably, CC One Blue exhibited a blue discoloration of the NaOCl solution after immersion at 60 °C, suggesting dissolution of its nano-coating and highlighting its increased susceptibility to corrosion.

Immersion in EDTA significantly reduced the CFR of NiTi files, adversely affecting their mechanical integrity and durability. These findings are consistent with Reinhard et al., who also reported the negative effects of EDTA on the fatigue resistance of NiTi rotary instruments (Reinhard et al. 1992). The detrimental impact of EDTA on CFR is attributed to the acidic pH of commercial EDTA solutions, which attack the metal surface to create stress concentrations and initiate cracks (Cai et al. 2017). The chemical interactions between EDTA and NiTi alloys result in corrosion and structural weaknesses, significantly compromising the fatigue resistance of the files (Erik and Özyürek 2019). Furthermore, Cai et al. observed that 17% EDTA markedly reduced the CFR of HEDM and M3 files, while 5.25% NaOCl exhibited no significant effects on these instruments (Cai et al. 2017). These results emphasize the stronger corrosive action of EDTA compared to NaOCl, exacerbated by its etching capabilities and the formation of stress concentration sites on metal surfaces.

The sequential application of NaOCl and EDTA produced the greatest impact on the CFR of the tested files, especially at higher temperatures. All file systems exhibited significantly lower TTF values under these conditions. These observations can be attributed to chemical interactions between NaOCl and EDTA, leading to the formation of calcium oxalate precipitates and other insoluble compounds. When combined, NaOCl oxidizes EDTA, triggering secondary chemical reactions. These precipitates may deposit on the surface of NiTi files, initiating corrosion processes that significantly impair their mechanical durability and structural integrity (Erik and Özyürek 2019; Ametrano et al. 2011). The SEM analysis corroborated the mechanical test results by revealing typical cyclic fatigue failure features, including crack origins and fatigue zones. Notably, micropitting was observed following immersion in 60 °C EDTA. Using NaOCl followed by EDTA produced pronounced surface irregularities and roughness, indicative of enhanced corrosion and degradation of the material. These fractographic changes are consistent with previous studies demonstrating the long-term impact of chemical influences on the surface structure of NiTi instruments (Pedullà et al. 2014). To mitigate these effects, frequent sequential use of NaOCl and EDTA should be avoided, and lower temperatures for both solutions should be employed to reduce the severity of these reactions and prolong instrument longevity. From a clinical perspective, our findings support current recommendations while also emphasizing the importance of controlling irrigant temperature. Although heated NaOCl enhances antimicrobial efficacy and tissue dissolution, its application should be carefully balanced against potential adverse effects on NiTi instruments, particularly in combination with EDTA. Clinicians should consider using EDTA at room temperature and limiting exposure times to minimize structural degradation. Additionally, given that Procodile Q demonstrated superior fatigue resistance, instruments that undergo machining after thermal treatment may offer advantages in cases requiring extensive irrigation.

The fragment length (FL) analysis revealed significant variations among the file systems and immersion conditions. Notably, EdgeOne R-Utopia and CC One Blue exhibited longer FL values, particularly under elevated temperatures (60 °C) and in EDTA solutions. These findings suggest that both thermal and chemical factors influence fracture behavior, likely by altering alloy properties and promoting changes in crack propagation dynamics. Longer fragments indicate a higher degree of plastic deformation before fracture, which may reflect reduced cyclic fatigue resistance under these conditions. Clinically, this highlights the importance of considering both irrigant choice and temperature in order to minimize adverse effects on instrument integrity.

The detrimental effects of frequent sequential NaOCl and EDTA exposure, particularly at elevated temperatures, highlight the need for cautious irrigant selection to preserve NiTi instrument longevity. Avoiding heated NaOCl followed by EDTA and limiting EDTA exposure time may help mitigate structural degradation.

Due to certain methodological constraints, the results of our study must be interpreted with caution. Despite using a standardized artificial root canal with a defined curvature, the model only partially represented the complexity and variability of natural root canal geometries, thereby limiting the clinical generalizability of the results. Another limitation was the relatively short immersion time of 5 min, which may not reflect real clinical contact times, particularly during prolonged treatments or repeated use of irrigating solutions. In addition, it should be noted that all instruments in this study were strictly used as single-use files, in accordance with current manufacturer recommendations.

The instruments in this study were strictly used as single-use files, in accordance with current manufacturer recommendations. This approach ensured consistency and eliminated the confounding effects of repeated sterilization or prior mechanical fatigue. However, it also represents an important limitation. In clinical practice, a single file is typically used for only one patient and remains in the canal for a very limited time. Therefore, even under extreme experimental conditions, the lowest time-to-fracture (TTF) values observed in this study (ranging from 34 to 102 s) may not directly correspond to realistic clinical scenarios. The influence of irrigating solutions on cyclic fatigue resistance, particularly under heated conditions, should thus be interpreted as a potential indicator of material susceptibility rather than a prediction of in vivo failure rates. Moreover, heated EDTA is rarely used in routine endodontic procedures, which further limits the direct clinical applicability.

All instruments were operated using the RECIPROC ALL mode of the VDW-Gold motor (VDW, Munich, Germany). This mode can be safely applied not only to Reciproc Blue but also to other reciprocating file systems with comparable kinematics. To ensure standardization and comparability across the experimental groups, all files were operated at 300 rpm under this setting, as described in the methods section. We acknowledge that some systems, such as R-Utopia and Procodile Q, may also be used with manufacturer-specific reciprocating programs; however, these settings are not available in the VDW-Gold motor, which represents a widely accepted reference device in laboratory testing. Since all tested files are approved for safe use in the RECIPROC ALL mode (150° CCW/30° CW), this standardized configuration was deliberately selected to minimize variability and ensure reproducibility of results. To gain a more complete understanding of how chemical and thermal factors influence the mechanical behavior of heat-treated NiTi files, future studies should consider longer exposure periods, additional irrigants, and clinical conditions.

## Conclusion

Within the limitations of the present study, heat-treated NiTi instruments were significantly influenced in their mechanical and metallurgical behavior by the chemical and thermal impact of irrigating solutions. Heated EDTA and the sequential application of NaOCl and EDTA produced the most pronounced reduction in CFR and caused visible surface degradation. Among the tested systems, Procodile Q consistently showed the highest CFR values under different immersion conditions. No significant differences were observed between high- and low-temperature heat-treated NiTi instruments with respect to CFR.

## Supplementary Information

Below is the link to the electronic supplementary material.

Supplementary Table 1. Results of the post-hoc pairwise comparisons for time to fracture (TTF) among all tested file types and immersion conditions. Data include mean differences, standard errors, and p-values (DOCX 83 KB)

## Data Availability

The data of this study are available from the corresponding author upon request.
