# Supplementary material for: Do irrigating solutions influence the cyclic fatigue resistance of heat-treated NiTi instruments? an in vitro study
Source: Saudi Dent J. 2026 Jan 23;38(2):10. doi: 10.1007/s44445-025-00093-0 (PMC12827828; doi:10.1007/s44445-025-00093-0)
Supplement: Supplementary file 1 — Supplementary Table 1. Results of the post-hoc pairwise comparisons for time to fracture (TTF) among all tested file types and immersion conditions. Data include mean differences, standard errors, and p-values (DOCX 83 KB) [file 44445_2025_93_MOESM1_ESM.docx]

**Influence of irrigating solutions on the cyclic fatigue resistance and surface integrity of heat-treated nickel-titanium instruments**

**Supplementary Table 1:** Post hoc analysis of time to fracture

| **Contrast** | **TTF (mean)** | **SE** | **P value** |
| --- | --- | --- | --- |
| CC One Control - Edge one Control | 82.633 | 16.856 | **0.001** |
| CC One Control - Procodile Q Control | -62.700 | 16.856 | 0.097 |
| CC One Control - Reciproc Blue Control | 4.133 | 15.526 | 1.0 |
| CC One Control - CC One EDTA_37°C | 36.800 | 15.869 | 0.951 |
| CC One Control - Edge one EDTA_37°C | 134.229 | 16.299 | **<0.001** |
| CC One Control - Procodile Q EDTA_37°C | -25.950 | 15.869 | 1.0 |
| CC One Control - Reciproc Blue EDTA_37°C | 33.800 | 15.869 | 0.985 |
| CC One Control - CC One EDTA_60°C | 56.800 | 16.856 | 0.25 |
| CC One Control - Edge one EDTA_60°C | 148.550 | 15.869 | **<0.001** |
| CC One Control - Procodile Q EDTA_60°C | 12.925 | 15.869 | 1.0 |
| CC One Control - Reciproc Blue EDTA_60°C | 48.133 | 15.526 | 0.441 |
| CC One Control - CC One Ethanol_37°C | 37.400 | 15.247 | 0.905 |
| CC One Control - Edge one Ethanol_37°C | 90.700 | 15.247 | **<0.001** |
| CC One Control - Procodile Q Ethanol_37°C | -67.000 | 15.247 | **0.009** |
| CC One Control - Reciproc Blue Ethanol_37°C | 11.700 | 15.247 | 1.0 |
| CC One Control - CC One H2O_37°C | -18.200 | 16.299 | 1.0 |
| CC One Control - Edge one H2O_37°C | 92.086 | 16.299 | **<0.001** |
| CC One Control - Procodile Q H2O_37°C | -90.533 | 15.526 | **<0.001** |
| CC One Control - Reciproc Blue H2O_37°C | -8.422 | 15.526 | 1.0 |
| CC One Control - CC One H2O_60°C | 4.086 | 16.299 | 1.0 |
| CC One Control - Edge one H2O_60°C | 94.175 | 15.869 | **<0.001** |
| CC One Control - Procodile Q H2O_60°C | -75.914 | 16.299 | **0.003** |
| CC One Control - Reciproc Blue H2O_60°C | 5.800 | 15.526 | 1.0 |
| CC One Control - CC One NaOCl_37°C | 33.925 | 15.869 | 0.984 |
| CC One Control - Edge one NaOCl_37°C | 116.800 | 17.605 | **<0.001** |
| CC One Control - Procodile Q NaOCl_37°C | -55.100 | 15.247 | 0.133 |
| CC One Control - Reciproc Blue NaOCl_37°C | 9.244 | 15.526 | 1.0 |
| CC One Control - CC One NaOCl_60°C | 124.086 | 16.299 | **<0.001** |
| CC One Control - Edge one NaOCl_60°C | 125.300 | 15.869 | **<0.001** |
| CC One Control - Procodile Q NaOCl_60°C | -28.700 | 15.869 | 0.999 |
| CC One Control - Reciproc Blue NaOCl_60°C | 17.600 | 17.605 | 1.0 |
| CC One Control - (CC One NaOCl+EDTA_37°C) | 93.900 | 15.247 | **<0.001** |
| CC One Control - (Edge one NaOCl+EDTA_37°C) | 123.700 | 15.247 | **<0.001** |
| CC One Control - (Procodile Q NaOCl+EDTA_37°C) | 26.900 | 15.247 | 1.0 |
| CC One Control - (Reciproc Blue NaOCl+EDTA_37°C) | 42.800 | 15.247 | 0.681 |
| CC One Control - (CC One NaOCl+EDTA_60°C) | 137.900 | 15.247 | **<0.001** |
| CC One Control - (Edge one NaOCl+EDTA_60°C) | 165.300 | 15.247 | **<0.001** |
| CC One Control - (Procodile Q NaOCl+EDTA_60°C) | 63.700 | 15.247 | 0.021 |
| CC One Control - (Reciproc Blue NaOCl+EDTA_60°C) | 75.500 | 15.247 | **<0.001** |
| Edge one Control - Procodile Q Control | -145.333 | 16.071 | **<0.001** |
| Edge one Control - Reciproc Blue Control | -78.500 | 14.671 | **<0.001** |
| Edge one Control - CC One EDTA_37°C | -45.833 | 15.033 | 0.482 |
| Edge one Control - Edge one EDTA_37°C | 51.595 | 15.487 | 0.274 |
| Edge one Control - Procodile Q EDTA_37°C | -108.583 | 15.033 | **<0.001** |
| Edge one Control - Reciproc Blue EDTA_37°C | -48.833 | 15.033 | 0.329 |
| Edge one Control - CC One EDTA_60°C | -25.833 | 16.071 | 1.0 |
| Edge one Control - Edge one EDTA_60°C | 65.917 | 15.033 | **0.01** |
| Edge one Control - Procodile Q EDTA_60°C | -69.708 | 15.033 | **0.003** |
| Edge one Control - Reciproc Blue EDTA_60°C | -34.500 | 14.671 | 0.942 |
| Edge one Control - CC One Ethanol_37°C | -45.233 | 14.375 | 0.404 |
| Edge one Control - Edge one Ethanol_37°C | 8.067 | 14.375 | 1.0 |
| Edge one Control - Procodile Q Ethanol_37°C | -149.633 | 14.375 | **<0.001** |
| Edge one Control - Reciproc Blue Ethanol_37°C | -70.933 | 14.375 | **<0.001** |
| Edge one Control - CC One H2O_37°C | -100.833 | 15.487 | **<0.001** |
| Edge one Control - Edge one H2O_37°C | 9.452 | 15.487 | 1.0 |
| Edge one Control - Procodile Q H2O_37°C | -173.167 | 14.671 | **<0.001** |
| Edge one Control - Reciproc Blue H2O_37°C | -91.056 | 14.671 | **<0.001** |
| Edge one Control - CC One H2O_60°C | -78.548 | 15.487 | **<0.001** |
| Edge one Control - Edge one H2O_60°C | 11.542 | 15.033 | 1.0 |
| Edge one Control - Procodile Q H2O_60°C | -158.548 | 15.487 | **<0.001** |
| Edge one Control - Reciproc Blue H2O_60°C | -76.833 | 14.671 | **<0.001** |
| Edge one Control - CC One NaOCl_37°C | -48.708 | 15.033 | 0.335 |
| Edge one Control - Edge one NaOCl_37°C | 34.167 | 16.856 | 0.993 |
| Edge one Control - Procodile Q NaOCl_37°C | -137.733 | 14.375 | **<0.001** |
| Edge one Control - Reciproc Blue NaOCl_37°C | -73.389 | 14.671 | **<0.001** |
| Edge one Control - CC One NaOCl_60°C | 41.452 | 15.487 | 0.779 |
| Edge one Control - Edge one NaOCl_60°C | 42.667 | 15.033 | 0.656 |
| Edge one Control - Procodile Q NaOCl_60°C | -111.333 | 15.033 | **<0.001** |
| Edge one Control - Reciproc Blue NaOCl_60°C | -65.033 | 16.856 | 0.063 |
| Edge one Control - (CC One NaOCl+EDTA_37°C) | 11.267 | 14.375 | 1.0 |
| Edge one Control - (Edge one NaOCl+EDTA_37°C) | 41.067 | 14.375 | 0.641 |
| Edge one Control - (Procodile Q NaOCl+EDTA_37°C) | -55.733 | 14.375 | 0.06 |
| Edge one Control - (Reciproc Blue NaOCl+EDTA_37°C) | -39.833 | 14.375 | 0.71 |
| Edge one Control - (CC One NaOCl+EDTA_60°C) | 55.267 | 14.375 | 0.066 |
| Edge one Control - (Edge one NaOCl+EDTA_60°C) | 82.667 | 14.375 | **<0.001** |
| Edge one Control - (Procodile Q NaOCl+EDTA_60°C) | -18.933 | 14.375 | 1.0 |
| Edge one Control - (Reciproc Blue NaOCl+EDTA_60°C) | -7.133 | 14.375 | 1.0 |
| Procodile Q Control - Reciproc Blue Control | 66.833 | 14.671 | **0.005** |
| Procodile Q Control - CC One EDTA_37°C | 99.500 | 15.033 | **<0.001** |
| Procodile Q Control - Edge one EDTA_37°C | 196.929 | 15.487 | **<0.001** |
| Procodile Q Control - Procodile Q EDTA_37°C | 36.750 | 15.033 | 0.908 |
| Procodile Q Control - Reciproc Blue EDTA_37°C | 96.500 | 15.033 | **<0.001** |
| Procodile Q Control - CC One EDTA_60°C | 119.500 | 16.071 | **<0.001** |
| Procodile Q Control - Edge one EDTA_60°C | 211.250 | 15.033 | **<0.001** |
| Procodile Q Control - Procodile Q EDTA_60°C | 75.625 | 15.033 | **<0.001** |
| Procodile Q Control - Reciproc Blue EDTA_60°C | 110.833 | 14.671 | **<0.001** |
| Procodile Q Control - CC One Ethanol_37°C | 100.100 | 14.375 | **<0.001** |
| Procodile Q Control - Edge one Ethanol_37°C | 153.400 | 14.375 | **<0.001** |
| Procodile Q Control - Procodile Q Ethanol_37°C | -4.300 | 14.375 | 1.0 |
| Procodile Q Control - Reciproc Blue Ethanol_37°C | 74.400 | 14.375 | **<0.001** |
| Procodile Q Control - CC One H2O_37°C | 44.500 | 15.487 | 0.628 |
| Procodile Q Control - Edge one H2O_37°C | 154.786 | 15.487 | **<0.001** |
| Procodile Q Control - Procodile Q H2O_37°C | -27.833 | 14.671 | 0.998 |
| Procodile Q Control - Reciproc Blue H2O_37°C | 54.278 | 14.671 | 0.103 |
| Procodile Q Control - CC One H2O_60°C | 66.786 | 15.487 | 0.013 |
| Procodile Q Control - Edge one H2O_60°C | 156.875 | 15.033 | **<0.001** |
| Procodile Q Control - Procodile Q H2O_60°C | -13.214 | 15.487 | 1.0 |
| Procodile Q Control - Reciproc Blue H2O_60°C | 68.500 | 14.671 | **0.003** |
| Procodile Q Control - CC One NaOCl_37°C | 96.625 | 15.033 | **<0.001** |
| Procodile Q Control - Edge one NaOCl_37°C | 179.500 | 16.856 | **<0.001** |
| Procodile Q Control - Procodile Q NaOCl_37°C | 7.600 | 14.375 | 1.0 |
| Procodile Q Control - Reciproc Blue NaOCl_37°C | 71.944 | 14.671 | **0.001** |
| Procodile Q Control - CC One NaOCl_60°C | 186.786 | 15.487 | **<0.001** |
| Procodile Q Control - Edge one NaOCl_60°C | 188.000 | 15.033 | **<0.001** |
| Procodile Q Control - Procodile Q NaOCl_60°C | 34.000 | 15.033 | 0.965 |
| Procodile Q Control - Reciproc Blue NaOCl_60°C | 80.300 | 16.856 | **0.002** |
| Procodile Q Control - (CC One NaOCl+EDTA_37°C) | 156.600 | 14.375 | **<0.001** |
| Procodile Q Control - (Edge one NaOCl+EDTA_37°C) | 186.400 | 14.375 | **<0.001** |
| Procodile Q Control - (Procodile Q NaOCl+EDTA_37°C) | 89.600 | 14.375 | **<0.001** |
| Procodile Q Control - (Reciproc Blue NaOCl+EDTA_37°C) | 105.500 | 14.375 | **<0.001** |
| Procodile Q Control - (CC One NaOCl+EDTA_60°C) | 200.600 | 14.375 | **<0.001** |
| Procodile Q Control - (Edge one NaOCl+EDTA_60°C) | 228.000 | 14.375 | **<0.001** |
| Procodile Q Control - (Procodile Q NaOCl+EDTA_60°C) | 126.400 | 14.375 | **<0.001** |
| Procodile Q Control - (Reciproc Blue NaOCl+EDTA_60°C) | 138.200 | 14.375 | **<0.001** |
| Reciproc Blue Control - CC One EDTA_37°C | 32.667 | 13.526 | 0.92 |
| Reciproc Blue Control - Edge one EDTA_37°C | 130.095 | 14.028 | **<0.001** |
| Reciproc Blue Control - Procodile Q EDTA_37°C | -30.083 | 13.526 | 0.972 |
| Reciproc Blue Control - Reciproc Blue EDTA_37°C | 29.667 | 13.526 | 0.977 |
| Reciproc Blue Control - CC One EDTA_60°C | 52.667 | 14.671 | 0.142 |
| Reciproc Blue Control - Edge one EDTA_60°C | 144.417 | 13.526 | **<0.001** |
| Reciproc Blue Control - Procodile Q EDTA_60°C | 8.792 | 13.526 | 1.0 |
| Reciproc Blue Control - Reciproc Blue EDTA_60°C | 44.000 | 13.122 | 0.26 |
| Reciproc Blue Control - CC One Ethanol_37°C | 33.267 | 12.790 | 0.828 |
| Reciproc Blue Control - Edge one Ethanol_37°C | 86.567 | 12.790 | **<0.001** |
| Reciproc Blue Control - Procodile Q Ethanol_37°C | -71.133 | 12.790 | **<0.001** |
| Reciproc Blue Control - Reciproc Blue Ethanol_37°C | 7.567 | 12.790 | 1.0 |
| Reciproc Blue Control - CC One H2O_37°C | -22.333 | 14.028 | 1.0 |
| Reciproc Blue Control - Edge one H2O_37°C | 87.952 | 14.028 | **<0.001** |
| Reciproc Blue Control - Procodile Q H2O_37°C | -94.667 | 13.122 | **<0.001** |
| Reciproc Blue Control - Reciproc Blue H2O_37°C | -12.556 | 13.122 | 1.0 |
| Reciproc Blue Control - CC One H2O_60°C | -0.048 | 14.028 | 1.0 |
| Reciproc Blue Control - Edge one H2O_60°C | 90.042 | 13.526 | **<0.001** |
| Reciproc Blue Control - Procodile Q H2O_60°C | -80.048 | 14.028 | **<0.001** |
| Reciproc Blue Control - Reciproc Blue H2O_60°C | 1.667 | 13.122 | 1.0 |
| Reciproc Blue Control - CC One NaOCl_37°C | 29.792 | 13.526 | 0.976 |
| Reciproc Blue Control - Edge one NaOCl_37°C | 112.667 | 15.526 | **<0.001** |
| Reciproc Blue Control - Procodile Q NaOCl_37°C | -59.233 | 12.790 | **0.003** |
| Reciproc Blue Control - Reciproc Blue NaOCl_37°C | 5.111 | 13.122 | 1.0 |
| Reciproc Blue Control - CC One NaOCl_60°C | 119.952 | 14.028 | **<0.001** |
| Reciproc Blue Control - Edge one NaOCl_60°C | 121.167 | 13.526 | **<0.001** |
| Reciproc Blue Control - Procodile Q NaOCl_60°C | -32.833 | 13.526 | 0.915 |
| Reciproc Blue Control - Reciproc Blue NaOCl_60°C | 13.467 | 15.526 | 1.0 |
| Reciproc Blue Control - (CC One NaOCl+EDTA_37°C) | 89.767 | 12.790 | **<0.001** |
| Reciproc Blue Control - (Edge one NaOCl+EDTA_37°C) | 119.567 | 12.790 | **<0.001** |
| Reciproc Blue Control - (Procodile Q NaOCl+EDTA_37°C) | 22.767 | 12.790 | 0.999 |
| Reciproc Blue Control - (Reciproc Blue NaOCl+EDTA_37°C) | 38.667 | 12.790 | 0.503 |
| Reciproc Blue Control - (CC One NaOCl+EDTA_60°C) | 133.767 | 12.790 | **<0.001** |
| Reciproc Blue Control - (Edge one NaOCl+EDTA_60°C) | 161.167 | 12.790 | **<0.001** |
| Reciproc Blue Control - (Procodile Q NaOCl+EDTA_60°C) | 59.567 | 12.790 | **0.003** |
| Reciproc Blue Control - (Reciproc Blue NaOCl+EDTA_60°C) | 71.367 | 12.790 | **<0.001** |
| CC One EDTA_37°C - Edge one EDTA_37°C | 97.429 | 14.407 | **<0.001** |
| CC One EDTA_37°C - Procodile Q EDTA_37°C | -62.750 | 13.918 | **0.006** |
| CC One EDTA_37°C - Reciproc Blue EDTA_37°C | -3.000 | 13.918 | 1.0 |
| CC One EDTA_37°C - CC One EDTA_60°C | 20.000 | 15.033 | 1.0 |
| CC One EDTA_37°C - Edge one EDTA_60°C | 111.750 | 13.918 | **<0.001** |
| CC One EDTA_37°C - Procodile Q EDTA_60°C | -23.875 | 13.918 | 1.0 |
| CC One EDTA_37°C - Reciproc Blue EDTA_60°C | 11.333 | 13.526 | 1.0 |
| CC One EDTA_37°C - CC One Ethanol_37°C | 0.600 | 13.204 | 1.0 |
| CC One EDTA_37°C - Edge one Ethanol_37°C | 53.900 | 13.204 | **0.03** |
| CC One EDTA_37°C - Procodile Q Ethanol_37°C | -103.800 | 13.204 | **<0.001** |
| CC One EDTA_37°C - Reciproc Blue Ethanol_37°C | -25.100 | 13.204 | 0.998 |
| CC One EDTA_37°C - CC One H2O_37°C | -55.000 | 14.407 | 0.072 |
| CC One EDTA_37°C - Edge one H2O_37°C | 55.286 | 14.407 | 0.068 |
| CC One EDTA_37°C - Procodile Q H2O_37°C | -127.333 | 13.526 | **<0.001** |
| CC One EDTA_37°C - Reciproc Blue H2O_37°C | -45.222 | 13.526 | 0.266 |
| CC One EDTA_37°C - CC One H2O_60°C | -32.714 | 14.407 | 0.963 |
| CC One EDTA_37°C - Edge one H2O_60°C | 57.375 | 13.918 | **0.026** |
| CC One EDTA_37°C - Procodile Q H2O_60°C | -112.714 | 14.407 | **<0.001** |
| CC One EDTA_37°C - Reciproc Blue H2O_60°C | -31.000 | 13.526 | 0.958 |
| CC One EDTA_37°C - CC One NaOCl_37°C | -2.875 | 13.918 | 1.0 |
| CC One EDTA_37°C - Edge one NaOCl_37°C | 80.000 | 15.869 | **<0.001** |
| CC One EDTA_37°C - Procodile Q NaOCl_37°C | -91.900 | 13.204 | **<0.001** |
| CC One EDTA_37°C - Reciproc Blue NaOCl_37°C | -27.556 | 13.526 | 0.993 |
| CC One EDTA_37°C - CC One NaOCl_60°C | 87.286 | 14.407 | **<0.001** |
| CC One EDTA_37°C - Edge one NaOCl_60°C | 88.500 | 13.918 | **<0.001** |
| CC One EDTA_37°C - Procodile Q NaOCl_60°C | -65.500 | 13.918 | **0.003** |
| CC One EDTA_37°C - Reciproc Blue NaOCl_60°C | -19.200 | 15.869 | 1.0 |
| CC One EDTA_37°C - (CC One NaOCl+EDTA_37°C) | 57.100 | 13.204 | **0.012** |
| CC One EDTA_37°C - (Edge one NaOCl+EDTA_37°C) | 86.900 | 13.204 | **<0.001** |
| CC One EDTA_37°C - (Procodile Q NaOCl+EDTA_37°C) | -9.900 | 13.204 | 1.0 |
| CC One EDTA_37°C - (Reciproc Blue NaOCl+EDTA_37°C) | 6.000 | 13.204 | 1.0 |
| CC One EDTA_37°C - (CC One NaOCl+EDTA_60°C) | 101.100 | 13.204 | **<0.001** |
| CC One EDTA_37°C - (Edge one NaOCl+EDTA_60°C) | 128.500 | 13.204 | **<0.001** |
| CC One EDTA_37°C - (Procodile Q NaOCl+EDTA_60°C) | 26.900 | 13.204 | 0.993 |
| CC One EDTA_37°C - (Reciproc Blue NaOCl+EDTA_60°C) | 38.700 | 13.204 | 0.58 |
| Edge one EDTA_37°C - Procodile Q EDTA_37°C | -160.179 | 14.407 | **<0.001** |
| Edge one EDTA_37°C - Reciproc Blue EDTA_37°C | -100.429 | 14.407 | **<0.001** |
| Edge one EDTA_37°C - CC One EDTA_60°C | -77.429 | 15.487 | **<0.001** |
| Edge one EDTA_37°C - Edge one EDTA_60°C | 14.321 | 14.407 | 1.0 |
| Edge one EDTA_37°C - Procodile Q EDTA_60°C | -121.304 | 14.407 | **<0.001** |
| Edge one EDTA_37°C - Reciproc Blue EDTA_60°C | -86.095 | 14.028 | **<0.001** |
| Edge one EDTA_37°C - CC One Ethanol_37°C | -96.829 | 13.718 | **<0.001** |
| Edge one EDTA_37°C - Edge one Ethanol_37°C | -43.529 | 13.718 | 0.384 |
| Edge one EDTA_37°C - Procodile Q Ethanol_37°C | -201.229 | 13.718 | **<0.001** |
| Edge one EDTA_37°C - Reciproc Blue Ethanol_37°C | -122.529 | 13.718 | **<0.001** |
| Edge one EDTA_37°C - CC One H2O_37°C | -152.429 | 14.879 | **<0.001** |
| Edge one EDTA_37°C - Edge one H2O_37°C | -42.143 | 14.879 | 0.661 |
| Edge one EDTA_37°C - Procodile Q H2O_37°C | -224.762 | 14.028 | **<0.001** |
| Edge one EDTA_37°C - Reciproc Blue H2O_37°C | -142.651 | 14.028 | **<0.001** |
| Edge one EDTA_37°C - CC One H2O_60°C | -130.143 | 14.879 | **<0.001** |
| Edge one EDTA_37°C - Edge one H2O_60°C | -40.054 | 14.407 | 0.702 |
| Edge one EDTA_37°C - Procodile Q H2O_60°C | -210.143 | 14.879 | **<0.001** |
| Edge one EDTA_37°C - Reciproc Blue H2O_60°C | -128.429 | 14.028 | **<0.001** |
| Edge one EDTA_37°C - CC One NaOCl_37°C | -100.304 | 14.407 | **<0.001** |
| Edge one EDTA_37°C - Edge one NaOCl_37°C | -17.429 | 16.299 | 1.0 |
| Edge one EDTA_37°C - Procodile Q NaOCl_37°C | -189.329 | 13.718 | **<0.001** |
| Edge one EDTA_37°C - Reciproc Blue NaOCl_37°C | -124.984 | 14.028 | **<0.001** |
| Edge one EDTA_37°C - CC One NaOCl_60°C | -10.143 | 14.879 | 1.0 |
| Edge one EDTA_37°C - Edge one NaOCl_60°C | -8.929 | 14.407 | 1.0 |
| Edge one EDTA_37°C - Procodile Q NaOCl_60°C | -162.929 | 14.407 | **<0.001** |
| Edge one EDTA_37°C - Reciproc Blue NaOCl_60°C | -116.629 | 16.299 | **<0.001** |
| Edge one EDTA_37°C - (CC One NaOCl+EDTA_37°C) | -40.329 | 13.718 | 0.573 |
| Edge one EDTA_37°C - (Edge one NaOCl+EDTA_37°C) | -10.529 | 13.718 | 1.0 |
| Edge one EDTA_37°C - (Procodile Q NaOCl+EDTA_37°C) | -107.329 | 13.718 | **<0.001** |
| Edge one EDTA_37°C - (Reciproc Blue NaOCl+EDTA_37°C) | -91.429 | 13.718 | **<0.001** |
| Edge one EDTA_37°C - (CC One NaOCl+EDTA_60°C) | 3.671 | 13.718 | 1.0 |
| Edge one EDTA_37°C - (Edge one NaOCl+EDTA_60°C) | 31.071 | 13.718 | 0.964 |
| Edge one EDTA_37°C - (Procodile Q NaOCl+EDTA_60°C) | -70.529 | 13.718 | **<0.001** |
| Edge one EDTA_37°C - (Reciproc Blue NaOCl+EDTA_60°C) | -58.729 | 13.718 | **0.014** |
| Procodile Q EDTA_37°C - Reciproc Blue EDTA_37°C | 59.750 | 13.918 | **0.014** |
| Procodile Q EDTA_37°C - CC One EDTA_60°C | 82.750 | 15.033 | **<0.001** |
| Procodile Q EDTA_37°C - Edge one EDTA_60°C | 174.500 | 13.918 | **<0.001** |
| Procodile Q EDTA_37°C - Procodile Q EDTA_60°C | 38.875 | 13.918 | 0.692 |
| Procodile Q EDTA_37°C - Reciproc Blue EDTA_60°C | 74.083 | 13.526 | **<0.001** |
| Procodile Q EDTA_37°C - CC One Ethanol_37°C | 63.350 | 13.204 | **0.002** |
| Procodile Q EDTA_37°C - Edge one Ethanol_37°C | 116.650 | 13.204 | **<0.001** |
| Procodile Q EDTA_37°C - Procodile Q Ethanol_37°C | -41.050 | 13.204 | 0.434 |
| Procodile Q EDTA_37°C - Reciproc Blue Ethanol_37°C | 37.650 | 13.204 | 0.646 |
| Procodile Q EDTA_37°C - CC One H2O_37°C | 7.750 | 14.407 | 1.0 |
| Procodile Q EDTA_37°C - Edge one H2O_37°C | 118.036 | 14.407 | **<0.001** |
| Procodile Q EDTA_37°C - Procodile Q H2O_37°C | -64.583 | 13.526 | **0.002** |
| Procodile Q EDTA_37°C - Reciproc Blue H2O_37°C | 17.528 | 13.526 | 1.0 |
| Procodile Q EDTA_37°C - CC One H2O_60°C | 30.036 | 14.407 | 0.989 |
| Procodile Q EDTA_37°C - Edge one H2O_60°C | 120.125 | 13.918 | **<0.001** |
| Procodile Q EDTA_37°C - Procodile Q H2O_60°C | -49.964 | 14.407 | 0.196 |
| Procodile Q EDTA_37°C - Reciproc Blue H2O_60°C | 31.750 | 13.526 | 0.943 |
| Procodile Q EDTA_37°C - CC One NaOCl_37°C | 59.875 | 13.918 | **0.013** |
| Procodile Q EDTA_37°C - Edge one NaOCl_37°C | 142.750 | 15.869 | **<0.001** |
| Procodile Q EDTA_37°C - Procodile Q NaOCl_37°C | -29.150 | 13.204 | 0.975 |
| Procodile Q EDTA_37°C - Reciproc Blue NaOCl_37°C | 35.194 | 13.526 | 0.827 |
| Procodile Q EDTA_37°C - CC One NaOCl_60°C | 150.036 | 14.407 | **<0.001** |
| Procodile Q EDTA_37°C - Edge one NaOCl_60°C | 151.250 | 13.918 | **<0.001** |
| Procodile Q EDTA_37°C - Procodile Q NaOCl_60°C | -2.750 | 13.918 | 1.0 |
| Procodile Q EDTA_37°C - Reciproc Blue NaOCl_60°C | 43.550 | 15.869 | 0.73 |
| Procodile Q EDTA_37°C - (CC One NaOCl+EDTA_37°C) | 119.850 | 13.204 | **<0.001** |
| Procodile Q EDTA_37°C - (Edge one NaOCl+EDTA_37°C) | 149.650 | 13.204 | **<0.001** |
| Procodile Q EDTA_37°C - (Procodile Q NaOCl+EDTA_37°C) | 52.850 | 13.204 | **0.039** |
| Procodile Q EDTA_37°C - (Reciproc Blue NaOCl+EDTA_37°C) | 68.750 | 13.204 | **<0.001** |
| Procodile Q EDTA_37°C - (CC One NaOCl+EDTA_60°C) | 163.850 | 13.204 | **<0.001** |
| Procodile Q EDTA_37°C - (Edge one NaOCl+EDTA_60°C) | 191.250 | 13.204 | **<0.001** |
| Procodile Q EDTA_37°C - (Procodile Q NaOCl+EDTA_60°C) | 89.650 | 13.204 | **<0.001** |
| Procodile Q EDTA_37°C - (Reciproc Blue NaOCl+EDTA_60°C) | 101.450 | 13.204 | **<0.001** |
| Reciproc Blue EDTA_37°C - CC One EDTA_60°C | 23.000 | 15.033 | 1.0 |
| Reciproc Blue EDTA_37°C - Edge one EDTA_60°C | 114.750 | 13.918 | **<0.001** |
| Reciproc Blue EDTA_37°C - Procodile Q EDTA_60°C | -20.875 | 13.918 | 1.0 |
| Reciproc Blue EDTA_37°C - Reciproc Blue EDTA_60°C | 14.333 | 13.526 | 1.0 |
| Reciproc Blue EDTA_37°C - CC One Ethanol_37°C | 3.600 | 13.204 | 1.0 |
| Reciproc Blue EDTA_37°C - Edge one Ethanol_37°C | 56.900 | 13.204 | **0.013** |
| Reciproc Blue EDTA_37°C - Procodile Q Ethanol_37°C | -100.800 | 13.204 | **<0.001** |
| Reciproc Blue EDTA_37°C - Reciproc Blue Ethanol_37°C | -22.100 | 13.204 | 1.0 |
| Reciproc Blue EDTA_37°C - CC One H2O_37°C | -52.000 | 14.407 | 0.134 |
| Reciproc Blue EDTA_37°C - Edge one H2O_37°C | 58.286 | 14.407 | **0.034** |
| Reciproc Blue EDTA_37°C - Procodile Q H2O_37°C | -124.333 | 13.526 | **<0.001** |
| Reciproc Blue EDTA_37°C - Reciproc Blue H2O_37°C | -42.222 | 13.526 | 0.424 |
| Reciproc Blue EDTA_37°C - CC One H2O_60°C | -29.714 | 14.407 | 0.991 |
| Reciproc Blue EDTA_37°C - Edge one H2O_60°C | 60.375 | 13.918 | **0.011** |
| Reciproc Blue EDTA_37°C - Procodile Q H2O_60°C | -109.714 | 14.407 | **<0.001** |
| Reciproc Blue EDTA_37°C - Reciproc Blue H2O_60°C | -28.000 | 13.526 | 0.991 |
| Reciproc Blue EDTA_37°C - CC One NaOCl_37°C | 0.125 | 13.918 | 1.0 |
| Reciproc Blue EDTA_37°C - Edge one NaOCl_37°C | 83.000 | 15.869 | **<0.001** |
| Reciproc Blue EDTA_37°C - Procodile Q NaOCl_37°C | -88.900 | 13.204 | **<0.001** |
| Reciproc Blue EDTA_37°C - Reciproc Blue NaOCl_37°C | -24.556 | 13.526 | 0.999 |
| Reciproc Blue EDTA_37°C - CC One NaOCl_60°C | 90.286 | 14.407 | **<0.001** |
| Reciproc Blue EDTA_37°C - Edge one NaOCl_60°C | 91.500 | 13.918 | **<0.001** |
| Reciproc Blue EDTA_37°C - Procodile Q NaOCl_60°C | -62.500 | 13.918 | **0.006** |
| Reciproc Blue EDTA_37°C - Reciproc Blue NaOCl_60°C | -16.200 | 15.869 | 1.0 |
| Reciproc Blue EDTA_37°C - (CC One NaOCl+EDTA_37°C) | 60.100 | 13.204 | **0.005** |
| Reciproc Blue EDTA_37°C - (Edge one NaOCl+EDTA_37°C) | 89.900 | 13.204 | **<0.001** |
| Reciproc Blue EDTA_37°C - (Procodile Q NaOCl+EDTA_37°C) | -6.900 | 13.204 | 1.0 |
| Reciproc Blue EDTA_37°C - (Reciproc Blue NaOCl+EDTA_37°C) | 9.000 | 13.204 | 1.0 |
| Reciproc Blue EDTA_37°C - (CC One NaOCl+EDTA_60°C) | 104.100 | 13.204 | **<0.001** |
| Reciproc Blue EDTA_37°C - (Edge one NaOCl+EDTA_60°C) | 131.500 | 13.204 | **<0.001** |
| Reciproc Blue EDTA_37°C - (Procodile Q NaOCl+EDTA_60°C) | 29.900 | 13.204 | 0.964 |
| Reciproc Blue EDTA_37°C - (Reciproc Blue NaOCl+EDTA_60°C) | 41.700 | 13.204 | 0.395 |
| CC One EDTA_60°C - Edge one EDTA_60°C | 91.750 | 15.033 | **<0.001** |
| CC One EDTA_60°C - Procodile Q EDTA_60°C | -43.875 | 15.033 | 0.59 |
| CC One EDTA_60°C - Reciproc Blue EDTA_60°C | -8.667 | 14.671 | 1.0 |
| CC One EDTA_60°C - CC One Ethanol_37°C | -19.400 | 14.375 | 1.0 |
| CC One EDTA_60°C - Edge one Ethanol_37°C | 33.900 | 14.375 | 0.94 |
| CC One EDTA_60°C - Procodile Q Ethanol_37°C | -123.800 | 14.375 | **<0.001** |
| CC One EDTA_60°C - Reciproc Blue Ethanol_37°C | -45.100 | 14.375 | 0.411 |
| CC One EDTA_60°C - CC One H2O_37°C | -75.000 | 15.487 | **0.001** |
| CC One EDTA_60°C - Edge one H2O_37°C | 35.286 | 15.487 | 0.961 |
| CC One EDTA_60°C - Procodile Q H2O_37°C | -147.333 | 14.671 | **<0.001** |
| CC One EDTA_60°C - Reciproc Blue H2O_37°C | -65.222 | 14.671 | **0.007** |
| CC One EDTA_60°C - CC One H2O_60°C | -52.714 | 15.487 | 0.231 |
| CC One EDTA_60°C - Edge one H2O_60°C | 37.375 | 15.033 | 0.89 |
| CC One EDTA_60°C - Procodile Q H2O_60°C | -132.714 | 15.487 | **<0.001** |
| CC One EDTA_60°C - Reciproc Blue H2O_60°C | -51.000 | 14.671 | 0.192 |
| CC One EDTA_60°C - CC One NaOCl_37°C | -22.875 | 15.033 | 1.0 |
| CC One EDTA_60°C - Edge one NaOCl_37°C | 60.000 | 16.856 | 0.154 |
| CC One EDTA_60°C - Procodile Q NaOCl_37°C | -111.900 | 14.375 | **<0.001** |
| CC One EDTA_60°C - Reciproc Blue NaOCl_37°C | -47.556 | 14.671 | 0.334 |
| CC One EDTA_60°C - CC One NaOCl_60°C | 67.286 | 15.487 | **0.011** |
| CC One EDTA_60°C - Edge one NaOCl_60°C | 68.500 | 15.033 | **0.005** |
| CC One EDTA_60°C - Procodile Q NaOCl_60°C | -85.500 | 15.033 | **<0.001** |
| CC One EDTA_60°C - Reciproc Blue NaOCl_60°C | -39.200 | 16.856 | 0.949 |
| CC One EDTA_60°C - (CC One NaOCl+EDTA_37°C) | 37.100 | 14.375 | 0.84 |
| CC One EDTA_60°C - (Edge one NaOCl+EDTA_37°C) | 66.900 | 14.375 | 0.003 |
| CC One EDTA_60°C - (Procodile Q NaOCl+EDTA_37°C) | -29.900 | 14.375 | 0.99 |
| CC One EDTA_60°C - (Reciproc Blue NaOCl+EDTA_37°C) | -14.000 | 14.375 | 1.0 |
| CC One EDTA_60°C - (CC One NaOCl+EDTA_60°C) | 81.100 | 14.375 | **<0.001** |
| CC One EDTA_60°C - (Edge one NaOCl+EDTA_60°C) | 108.500 | 14.375 | **<0.001** |
| CC One EDTA_60°C - (Procodile Q NaOCl+EDTA_60°C) | 6.900 | 14.375 | 1.0 |
| CC One EDTA_60°C - (Reciproc Blue NaOCl+EDTA_60°C) | 18.700 | 14.375 | 1.0 |
| Edge one EDTA_60°C - Procodile Q EDTA_60°C | -135.625 | 13.918 | **<0.001** |
| Edge one EDTA_60°C - Reciproc Blue EDTA_60°C | -100.417 | 13.526 | **<0.001** |
| Edge one EDTA_60°C - CC One Ethanol_37°C | -111.150 | 13.204 | **<0.001** |
| Edge one EDTA_60°C - Edge one Ethanol_37°C | -57.850 | 13.204 | **0.01** |
| Edge one EDTA_60°C - Procodile Q Ethanol_37°C | -215.550 | 13.204 | **<0.001** |
| Edge one EDTA_60°C - Reciproc Blue Ethanol_37°C | -136.850 | 13.204 | **<0.001** |
| Edge one EDTA_60°C - CC One H2O_37°C | -166.750 | 14.407 | **<0.001** |
| Edge one EDTA_60°C - Edge one H2O_37°C | -56.464 | 14.407 | 0.052 |
| Edge one EDTA_60°C - Procodile Q H2O_37°C | -239.083 | 13.526 | **<0.001** |
| Edge one EDTA_60°C - Reciproc Blue H2O_37°C | -156.972 | 13.526 | **<0.001** |
| Edge one EDTA_60°C - CC One H2O_60°C | -144.464 | 14.407 | **<0.001** |
| Edge one EDTA_60°C - Edge one H2O_60°C | -54.375 | 13.918 | 0.054 |
| Edge one EDTA_60°C - Procodile Q H2O_60°C | -224.464 | 14.407 | **<0.001** |
| Edge one EDTA_60°C - Reciproc Blue H2O_60°C | -142.750 | 13.526 | **<0.001** |
| Edge one EDTA_60°C - CC One NaOCl_37°C | -114.625 | 13.918 | **<0.001** |
| Edge one EDTA_60°C - Edge one NaOCl_37°C | -31.750 | 15.869 | 0.995 |
| Edge one EDTA_60°C - Procodile Q NaOCl_37°C | -203.650 | 13.204 | **<0.001** |
| Edge one EDTA_60°C - Reciproc Blue NaOCl_37°C | -139.306 | 13.526 | **<0.001** |
| Edge one EDTA_60°C - CC One NaOCl_60°C | -24.464 | 14.407 | 1.0 |
| Edge one EDTA_60°C - Edge one NaOCl_60°C | -23.250 | 13.918 | 1.0 |
| Edge one EDTA_60°C - Procodile Q NaOCl_60°C | -177.250 | 13.918 | **<0.001** |
| Edge one EDTA_60°C - Reciproc Blue NaOCl_60°C | -130.950 | 15.869 | **<0.001** |
| Edge one EDTA_60°C - (CC One NaOCl+EDTA_37°C) | -54.650 | 13.204 | **0.024** |
| Edge one EDTA_60°C - (Edge one NaOCl+EDTA_37°C) | -24.850 | 13.204 | 0.998 |
| Edge one EDTA_60°C - (Procodile Q NaOCl+EDTA_37°C) | -121.650 | 13.204 | **<0.001** |
| Edge one EDTA_60°C - (Reciproc Blue NaOCl+EDTA_37°C) | -105.750 | 13.204 | **<0.001** |
| Edge one EDTA_60°C - (CC One NaOCl+EDTA_60°C) | -10.650 | 13.204 | 1.0 |
| Edge one EDTA_60°C - (Edge one NaOCl+EDTA_60°C) | 16.750 | 13.204 | 1.0 |
| Edge one EDTA_60°C - (Procodile Q NaOCl+EDTA_60°C) | -84.850 | 13.204 | **<0.001** |
| Edge one EDTA_60°C - (Reciproc Blue NaOCl+EDTA_60°C) | -73.050 | 13.204 | **<0.001** |
| Procodile Q EDTA_60°C - Reciproc Blue EDTA_60°C | 35.208 | 13.526 | 0.827 |
| Procodile Q EDTA_60°C - CC One Ethanol_37°C | 24.475 | 13.204 | 0.999 |
| Procodile Q EDTA_60°C - Edge one Ethanol_37°C | 77.775 | 13.204 | **<0.001** |
| Procodile Q EDTA_60°C - Procodile Q Ethanol_37°C | -79.925 | 13.204 | **<0.001** |
| Procodile Q EDTA_60°C - Reciproc Blue Ethanol_37°C | -1.225 | 13.204 | 1.0 |
| Procodile Q EDTA_60°C - CC One H2O_37°C | -31.125 | 14.407 | 0.982 |
| Procodile Q EDTA_60°C - Edge one H2O_37°C | 79.161 | 14.407 | **<0.001** |
| Procodile Q EDTA_60°C - Procodile Q H2O_37°C | -103.458 | 13.526 | **<0.001** |
| Procodile Q EDTA_60°C - Reciproc Blue H2O_37°C | -21.347 | 13.526 | 1.0 |
| Procodile Q EDTA_60°C - CC One H2O_60°C | -8.839 | 14.407 | 1.0 |
| Procodile Q EDTA_60°C - Edge one H2O_60°C | 81.250 | 13.918 | **<0.001** |
| Procodile Q EDTA_60°C - Procodile Q H2O_60°C | -88.839 | 14.407 | **<0.001** |
| Procodile Q EDTA_60°C - Reciproc Blue H2O_60°C | -7.125 | 13.526 | 1.0 |
| Procodile Q EDTA_60°C - CC One NaOCl_37°C | 21.000 | 13.918 | 1.0 |
| Procodile Q EDTA_60°C - Edge one NaOCl_37°C | 103.875 | 15.869 | **<0.001** |
| Procodile Q EDTA_60°C - Procodile Q NaOCl_37°C | -68.025 | 13.204 | **<0.001** |
| Procodile Q EDTA_60°C - Reciproc Blue NaOCl_37°C | -3.681 | 13.526 | 1.0 |
| Procodile Q EDTA_60°C - CC One NaOCl_60°C | 111.161 | 14.407 | **<0.001** |
| Procodile Q EDTA_60°C - Edge one NaOCl_60°C | 112.375 | 13.918 | **<0.001** |
| Procodile Q EDTA_60°C - Procodile Q NaOCl_60°C | -41.625 | 13.918 | 0.53 |
| Procodile Q EDTA_60°C - Reciproc Blue NaOCl_60°C | 4.675 | 15.869 | 1.0 |
| Procodile Q EDTA_60°C - (CC One NaOCl+EDTA_37°C) | 80.975 | 13.204 | **<0.001** |
| Procodile Q EDTA_60°C - (Edge one NaOCl+EDTA_37°C) | 110.775 | 13.204 | **<0.001** |
| Procodile Q EDTA_60°C - (Procodile Q NaOCl+EDTA_37°C) | 13.975 | 13.204 | 1.0 |
| Procodile Q EDTA_60°C - (Reciproc Blue NaOCl+EDTA_37°C) | 29.875 | 13.204 | 0.965 |
| Procodile Q EDTA_60°C - (CC One NaOCl+EDTA_60°C) | 124.975 | 13.204 | **<0.001** |
| Procodile Q EDTA_60°C - (Edge one NaOCl+EDTA_60°C) | 152.375 | 13.204 | **<0.001** |
| Procodile Q EDTA_60°C - (Procodile Q NaOCl+EDTA_60°C) | 50.775 | 13.204 | 0.066 |
| Procodile Q EDTA_60°C - (Reciproc Blue NaOCl+EDTA_60°C) | 62.575 | 13.204 | **0.002** |
| Reciproc Blue EDTA_60°C - CC One Ethanol_37°C | -10.733 | 12.790 | 1.0 |
| Reciproc Blue EDTA_60°C - Edge one Ethanol_37°C | 42.567 | 12.790 | 0.276 |
| Reciproc Blue EDTA_60°C - Procodile Q Ethanol_37°C | -115.133 | 12.790 | **<0.001** |
| Reciproc Blue EDTA_60°C - Reciproc Blue Ethanol_37°C | -36.433 | 12.790 | 0.648 |
| Reciproc Blue EDTA_60°C - CC One H2O_37°C | -66.333 | 14.028 | **0.002** |
| Reciproc Blue EDTA_60°C - Edge one H2O_37°C | 43.952 | 14.028 | 0.414 |
| Reciproc Blue EDTA_60°C - Procodile Q H2O_37°C | -138.667 | 13.122 | **<0.001** |
| Reciproc Blue EDTA_60°C - Reciproc Blue H2O_37°C | -56.556 | 13.122 | **0.013** |
| Reciproc Blue EDTA_60°C - CC One H2O_60°C | -44.048 | 14.028 | 0.409 |
| Reciproc Blue EDTA_60°C - Edge one H2O_60°C | 46.042 | 13.526 | 0.231 |
| Reciproc Blue EDTA_60°C - Procodile Q H2O_60°C | -124.048 | 14.028 | **<0.001** |
| Reciproc Blue EDTA_60°C - Reciproc Blue H2O_60°C | -42.333 | 13.122 | 0.345 |
| Reciproc Blue EDTA_60°C - CC One NaOCl_37°C | -14.208 | 13.526 | 1.0 |
| Reciproc Blue EDTA_60°C - Edge one NaOCl_37°C | 68.667 | 15.526 | **0.008** |
| Reciproc Blue EDTA_60°C - Procodile Q NaOCl_37°C | -103.233 | 12.790 | **<0.001** |
| Reciproc Blue EDTA_60°C - Reciproc Blue NaOCl_37°C | -38.889 | 13.122 | 0.553 |
| Reciproc Blue EDTA_60°C - CC One NaOCl_60°C | 75.952 | 14.028 | **<0.001** |
| Reciproc Blue EDTA_60°C - Edge one NaOCl_60°C | 77.167 | 13.526 | **<0.001** |
| Reciproc Blue EDTA_60°C - Procodile Q NaOCl_60°C | -76.833 | 13.526 | **<0.001** |
| Reciproc Blue EDTA_60°C - Reciproc Blue NaOCl_60°C | -30.533 | 15.526 | 0.996 |
| Reciproc Blue EDTA_60°C - (CC One NaOCl+EDTA_37°C) | 45.767 | 12.790 | 0.147 |
| Reciproc Blue EDTA_60°C - (Edge one NaOCl+EDTA_37°C) | 75.567 | 12.790 | **<0.001** |
| Reciproc Blue EDTA_60°C - (Procodile Q NaOCl+EDTA_37°C) | -21.233 | 12.790 | 1.0 |
| Reciproc Blue EDTA_60°C - (Reciproc Blue NaOCl+EDTA_37°C) | -5.333 | 12.790 | 1.0 |
| Reciproc Blue EDTA_60°C - (CC One NaOCl+EDTA_60°C) | 89.767 | 12.790 | **<0.001** |
| Reciproc Blue EDTA_60°C - (Edge one NaOCl+EDTA_60°C) | 117.167 | 12.790 | **<0.001** |
| Reciproc Blue EDTA_60°C - (Procodile Q NaOCl+EDTA_60°C) | 15.567 | 12.790 | 1.0 |
| Reciproc Blue EDTA_60°C - (Reciproc Blue NaOCl+EDTA_60°C) | 27.367 | 12.790 | 0.984 |
| CC One Ethanol_37°C - Edge one Ethanol_37°C | 53.300 | 12.449 | **0.014** |
| CC One Ethanol_37°C - Procodile Q Ethanol_37°C | -104.400 | 12.449 | **<0.001** |
| CC One Ethanol_37°C - Reciproc Blue Ethanol_37°C | -25.700 | 12.449 | 0.991 |
| CC One Ethanol_37°C - CC One H2O_37°C | -55.600 | 13.718 | **0.033** |
| CC One Ethanol_37°C - Edge one H2O_37°C | 54.686 | 13.718 | **0.041** |
| CC One Ethanol_37°C - Procodile Q H2O_37°C | -127.933 | 12.790 | **<0.001** |
| CC One Ethanol_37°C - Reciproc Blue H2O_37°C | -45.822 | 12.790 | 0.145 |
| CC One Ethanol_37°C - CC One H2O_60°C | -33.314 | 13.718 | 0.915 |
| CC One Ethanol_37°C - Edge one H2O_60°C | 56.775 | 13.204 | **0.013** |
| CC One Ethanol_37°C - Procodile Q H2O_60°C | -113.314 | 13.718 | **<0.001** |
| CC One Ethanol_37°C - Reciproc Blue H2O_60°C | -31.600 | 12.790 | 0.897 |
| CC One Ethanol_37°C - CC One NaOCl_37°C | -3.475 | 13.204 | 1.0 |
| CC One Ethanol_37°C - Edge one NaOCl_37°C | 79.400 | 15.247 | **<0.001** |
| CC One Ethanol_37°C - Procodile Q NaOCl_37°C | -92.500 | 12.449 | **<0.001** |
| CC One Ethanol_37°C - Reciproc Blue NaOCl_37°C | -28.156 | 12.790 | 0.976 |
| CC One Ethanol_37°C - CC One NaOCl_60°C | 86.686 | 13.718 | **<0.001** |
| CC One Ethanol_37°C - Edge one NaOCl_60°C | 87.900 | 13.204 | **<0.001** |
| CC One Ethanol_37°C - Procodile Q NaOCl_60°C | -66.100 | 13.204 | **<0.001** |
| CC One Ethanol_37°C - Reciproc Blue NaOCl_60°C | -19.800 | 15.247 | 1.0 |
| CC One Ethanol_37°C - (CC One NaOCl+EDTA_37°C) | 56.500 | 12.449 | **0.005** |
| CC One Ethanol_37°C - (Edge one NaOCl+EDTA_37°C) | 86.300 | 12.449 | **<0.001** |
| CC One Ethanol_37°C - (Procodile Q NaOCl+EDTA_37°C) | -10.500 | 12.449 | 1.0 |
| CC One Ethanol_37°C - (Reciproc Blue NaOCl+EDTA_37°C) | 5.400 | 12.449 | 1.0 |
| CC One Ethanol_37°C - (CC One NaOCl+EDTA_60°C) | 100.500 | 12.449 | **<0.001** |
| CC One Ethanol_37°C - (Edge one NaOCl+EDTA_60°C) | 127.900 | 12.449 | **<0.001** |
| CC One Ethanol_37°C - (Procodile Q NaOCl+EDTA_60°C) | 26.300 | 12.449 | 0.987 |
| CC One Ethanol_37°C - (Reciproc Blue NaOCl+EDTA_60°C) | 38.100 | 12.449 | 0.472 |
| Edge one Ethanol_37°C - Procodile Q Ethanol_37°C | -157.700 | 12.449 | **<0.001** |
| Edge one Ethanol_37°C - Reciproc Blue Ethanol_37°C | -79.000 | 12.449 | **<0.001** |
| Edge one Ethanol_37°C - CC One H2O_37°C | -108.900 | 13.718 | **<0.001** |
| Edge one Ethanol_37°C - Edge one H2O_37°C | 1.386 | 13.718 | 1.0 |
| Edge one Ethanol_37°C - Procodile Q H2O_37°C | -181.233 | 12.790 | **<0.001** |
| Edge one Ethanol_37°C - Reciproc Blue H2O_37°C | -99.122 | 12.790 | **<0.001** |
| Edge one Ethanol_37°C - CC One H2O_60°C | -86.614 | 13.718 | **<0.001** |
| Edge one Ethanol_37°C - Edge one H2O_60°C | 3.475 | 13.204 | 1.0 |
| Edge one Ethanol_37°C - Procodile Q H2O_60°C | -166.614 | 13.718 | **<0.001** |
| Edge one Ethanol_37°C - Reciproc Blue H2O_60°C | -84.900 | 12.790 | **<0.001** |
| Edge one Ethanol_37°C - CC One NaOCl_37°C | -56.775 | 13.204 | **0.013** |
| Edge one Ethanol_37°C - Edge one NaOCl_37°C | 26.100 | 15.247 | 1.0 |
| Edge one Ethanol_37°C - Procodile Q NaOCl_37°C | -145.800 | 12.449 | **<0.001** |
| Edge one Ethanol_37°C - Reciproc Blue NaOCl_37°C | -81.456 | 12.790 | **<0.001** |
| Edge one Ethanol_37°C - CC One NaOCl_60°C | 33.386 | 13.718 | 0.913 |
| Edge one Ethanol_37°C - Edge one NaOCl_60°C | 34.600 | 13.204 | 0.816 |
| Edge one Ethanol_37°C - Procodile Q NaOCl_60°C | -119.400 | 13.204 | **<0.001** |
| Edge one Ethanol_37°C - Reciproc Blue NaOCl_60°C | -73.100 | 15.247 | **0.002** |
| Edge one Ethanol_37°C - (CC One NaOCl+EDTA_37°C) | 3.200 | 12.449 | 1.0 |
| Edge one Ethanol_37°C - (Edge one NaOCl+EDTA_37°C) | 33.000 | 12.449 | 0.796 |
| Edge one Ethanol_37°C - (Procodile Q NaOCl+EDTA_37°C) | -63.800 | 12.449 | **<0.001** |
| Edge one Ethanol_37°C - (Reciproc Blue NaOCl+EDTA_37°C) | -47.900 | 12.449 | 0.065 |
| Edge one Ethanol_37°C - (CC One NaOCl+EDTA_60°C) | 47.200 | 12.449 | 0.078 |
| Edge one Ethanol_37°C - (Edge one NaOCl+EDTA_60°C) | 74.600 | 12.449 | **<0.001** |
| Edge one Ethanol_37°C - (Procodile Q NaOCl+EDTA_60°C) | -27.000 | 12.449 | 0.98 |
| Edge one Ethanol_37°C - (Reciproc Blue NaOCl+EDTA_60°C) | -15.200 | 12.449 | 1.0 |
| Procodile Q Ethanol_37°C - Reciproc Blue Ethanol_37°C | 78.700 | 12.449 | **<0.001** |
| Procodile Q Ethanol_37°C - CC One H2O_37°C | 48.800 | 13.718 | 0.155 |
| Procodile Q Ethanol_37°C - Edge one H2O_37°C | 159.086 | 13.718 | **<0.001** |
| Procodile Q Ethanol_37°C - Procodile Q H2O_37°C | -23.533 | 12.790 | 0.999 |
| Procodile Q Ethanol_37°C - Reciproc Blue H2O_37°C | 58.578 | 12.790 | **0.004** |
| Procodile Q Ethanol_37°C - CC One H2O_60°C | 71.086 | 13.718 | **<0.001** |
| Procodile Q Ethanol_37°C - Edge one H2O_60°C | 161.175 | 13.204 | **<0.001** |
| Procodile Q Ethanol_37°C - Procodile Q H2O_60°C | -8.914 | 13.718 | 1.0 |
| Procodile Q Ethanol_37°C - Reciproc Blue H2O_60°C | 72.800 | 12.790 | **<0.001** |
| Procodile Q Ethanol_37°C - CC One NaOCl_37°C | 100.925 | 13.204 | **<0.001** |
| Procodile Q Ethanol_37°C - Edge one NaOCl_37°C | 183.800 | 15.247 | **<0.001** |
| Procodile Q Ethanol_37°C - Procodile Q NaOCl_37°C | 11.900 | 12.449 | 1.0 |
| Procodile Q Ethanol_37°C - Reciproc Blue NaOCl_37°C | 76.244 | 12.790 | **<0.001** |
| Procodile Q Ethanol_37°C - CC One NaOCl_60°C | 191.086 | 13.718 | **<0.001** |
| Procodile Q Ethanol_37°C - Edge one NaOCl_60°C | 192.300 | 13.204 | **<0.001** |
| Procodile Q Ethanol_37°C - Procodile Q NaOCl_60°C | 38.300 | 13.204 | 0.605 |
| Procodile Q Ethanol_37°C - Reciproc Blue NaOCl_60°C | 84.600 | 15.247 | **<0.001** |
| Procodile Q Ethanol_37°C - (CC One NaOCl+EDTA_37°C) | 160.900 | 12.449 | **<0.001** |
| Procodile Q Ethanol_37°C - (Edge one NaOCl+EDTA_37°C) | 190.700 | 12.449 | **<0.001** |
| Procodile Q Ethanol_37°C - (Procodile Q NaOCl+EDTA_37°C) | 93.900 | 12.449 | **<0.001** |
| Procodile Q Ethanol_37°C - (Reciproc Blue NaOCl+EDTA_37°C) | 109.800 | 12.449 | **<0.001** |
| Procodile Q Ethanol_37°C - (CC One NaOCl+EDTA_60°C) | 204.900 | 12.449 | **<0.001** |
| Procodile Q Ethanol_37°C - (Edge one NaOCl+EDTA_60°C) | 232.300 | 12.449 | **<0.001** |
| Procodile Q Ethanol_37°C - (Procodile Q NaOCl+EDTA_60°C) | 130.700 | 12.449 | **<0.001** |
| Procodile Q Ethanol_37°C - (Reciproc Blue NaOCl+EDTA_60°C) | 142.500 | 12.449 | **<0.001** |
| Reciproc Blue Ethanol_37°C - CC One H2O_37°C | -29.900 | 13.718 | 0.979 |
| Reciproc Blue Ethanol_37°C - Edge one H2O_37°C | 80.386 | 13.718 | **<0.001** |
| Reciproc Blue Ethanol_37°C - Procodile Q H2O_37°C | -102.233 | 12.790 | **<0.001** |
| Reciproc Blue Ethanol_37°C - Reciproc Blue H2O_37°C | -20.122 | 12.790 | 1.0 |
| Reciproc Blue Ethanol_37°C - CC One H2O_60°C | -7.614 | 13.718 | 1.0 |
| Reciproc Blue Ethanol_37°C - Edge one H2O_60°C | 82.475 | 13.204 | **<0.001** |
| Reciproc Blue Ethanol_37°C - Procodile Q H2O_60°C | -87.614 | 13.718 | **<0.001** |
| Reciproc Blue Ethanol_37°C - Reciproc Blue H2O_60°C | -5.900 | 12.790 | 1.0 |
| Reciproc Blue Ethanol_37°C - CC One NaOCl_37°C | 22.225 | 13.204 | 1.0 |
| Reciproc Blue Ethanol_37°C - Edge one NaOCl_37°C | 105.100 | 15.247 | **<0.001** |
| Reciproc Blue Ethanol_37°C - Procodile Q NaOCl_37°C | -66.800 | 12.449 | **<0.001** |
| Reciproc Blue Ethanol_37°C - Reciproc Blue NaOCl_37°C | -2.456 | 12.790 | 1.0 |
| Reciproc Blue Ethanol_37°C - CC One NaOCl_60°C | 112.386 | 13.718 | **<0.001** |
| Reciproc Blue Ethanol_37°C - Edge one NaOCl_60°C | 113.600 | 13.204 | **<0.001** |
| Reciproc Blue Ethanol_37°C - Procodile Q NaOCl_60°C | -40.400 | 13.204 | 0.473 |
| Reciproc Blue Ethanol_37°C - Reciproc Blue NaOCl_60°C | 5.900 | 15.247 | 1.0 |
| Reciproc Blue Ethanol_37°C - (CC One NaOCl+EDTA_37°C) | 82.200 | 12.449 | **<0.001** |
| Reciproc Blue Ethanol_37°C - (Edge one NaOCl+EDTA_37°C) | 112.000 | 12.449 | **<0.001** |
| Reciproc Blue Ethanol_37°C - (Procodile Q NaOCl+EDTA_37°C) | 15.200 | 12.449 | 1.0 |
| Reciproc Blue Ethanol_37°C - (Reciproc Blue NaOCl+EDTA_37°C) | 31.100 | 12.449 | 0.884 |
| Reciproc Blue Ethanol_37°C - (CC One NaOCl+EDTA_60°C) | 126.200 | 12.449 | **<0.001** |
| Reciproc Blue Ethanol_37°C - (Edge one NaOCl+EDTA_60°C) | 153.600 | 12.449 | **<0.001** |
| Reciproc Blue Ethanol_37°C - (Procodile Q NaOCl+EDTA_60°C) | 52.000 | 12.449 | **0.021** |
| Reciproc Blue Ethanol_37°C - (Reciproc Blue NaOCl+EDTA_60°C) | 63.800 | 12.449 | **<0.001** |
| CC One H2O_37°C - Edge one H2O_37°C | 110.286 | 14.879 | **<0.001** |
| CC One H2O_37°C - Procodile Q H2O_37°C | -72.333 | 14.028 | **<0.001** |
| CC One H2O_37°C - Reciproc Blue H2O_37°C | 9.778 | 14.028 | 1.0 |
| CC One H2O_37°C - CC One H2O_60°C | 22.286 | 14.879 | 1.0 |
| CC One H2O_37°C - Edge one H2O_60°C | 112.375 | 14.407 | **<0.001** |
| CC One H2O_37°C - Procodile Q H2O_60°C | -57.714 | 14.879 | 0.059 |
| CC One H2O_37°C - Reciproc Blue H2O_60°C | 24.000 | 14.028 | 1.0 |
| CC One H2O_37°C - CC One NaOCl_37°C | 52.125 | 14.407 | 0.131 |
| CC One H2O_37°C - Edge one NaOCl_37°C | 135.000 | 16.299 | **<0.001** |
| CC One H2O_37°C - Procodile Q NaOCl_37°C | -36.900 | 13.718 | 0.77 |
| CC One H2O_37°C - Reciproc Blue NaOCl_37°C | 27.444 | 14.028 | 0.996 |
| CC One H2O_37°C - CC One NaOCl_60°C | 142.286 | 14.879 | **<0.001** |
| CC One H2O_37°C - Edge one NaOCl_60°C | 143.500 | 14.407 | **<0.001** |
| CC One H2O_37°C - Procodile Q NaOCl_60°C | -10.500 | 14.407 | 1.0 |
| CC One H2O_37°C - Reciproc Blue NaOCl_60°C | 35.800 | 16.299 | 0.977 |
| CC One H2O_37°C - (CC One NaOCl+EDTA_37°C) | 112.100 | 13.718 | **<0.001** |
| CC One H2O_37°C - (Edge one NaOCl+EDTA_37°C) | 141.900 | 13.718 | **<0.001** |
| CC One H2O_37°C - (Procodile Q NaOCl+EDTA_37°C) | 45.100 | 13.718 | 0.302 |
| CC One H2O_37°C - (Reciproc Blue NaOCl+EDTA_37°C) | 61.000 | 13.718 | **0.007** |
| CC One H2O_37°C - (CC One NaOCl+EDTA_60°C) | 156.100 | 13.718 | **<0.001** |
| CC One H2O_37°C - (Edge one NaOCl+EDTA_60°C) | 183.500 | 13.718 | **<0.001** |
| CC One H2O_37°C - (Procodile Q NaOCl+EDTA_60°C) | 81.900 | 13.718 | **<0.001** |
| CC One H2O_37°C - (Reciproc Blue NaOCl+EDTA_60°C) | 93.700 | 13.718 | **<0.001** |
| Edge one H2O_37°C - Procodile Q H2O_37°C | -182.619 | 14.028 | **<0.001** |
| Edge one H2O_37°C - Reciproc Blue H2O_37°C | -100.508 | 14.028 | **<0.001** |
| Edge one H2O_37°C - CC One H2O_60°C | -88.000 | 14.879 | **<0.001** |
| Edge one H2O_37°C - Edge one H2O_60°C | 2.089 | 14.407 | 1.0 |
| Edge one H2O_37°C - Procodile Q H2O_60°C | -168.000 | 14.879 | **<0.001** |
| Edge one H2O_37°C - Reciproc Blue H2O_60°C | -86.286 | 14.028 | **<0.001** |
| Edge one H2O_37°C - CC One NaOCl_37°C | -58.161 | 14.407 | 0.035 |
| Edge one H2O_37°C - Edge one NaOCl_37°C | 24.714 | 16.299 | 1.0 |
| Edge one H2O_37°C - Procodile Q NaOCl_37°C | -147.186 | 13.718 | **<0.001** |
| Edge one H2O_37°C - Reciproc Blue NaOCl_37°C | -82.841 | 14.028 | **<0.001** |
| Edge one H2O_37°C - CC One NaOCl_60°C | 32.000 | 14.879 | 0.983 |
| Edge one H2O_37°C - Edge one NaOCl_60°C | 33.214 | 14.407 | 0.955 |
| Edge one H2O_37°C - Procodile Q NaOCl_60°C | -120.786 | 14.407 | **<0.001** |
| Edge one H2O_37°C - Reciproc Blue NaOCl_60°C | -74.486 | 16.299 | **0.004** |
| Edge one H2O_37°C - (CC One NaOCl+EDTA_37°C) | 1.814 | 13.718 | 1.0 |
| Edge one H2O_37°C - (Edge one NaOCl+EDTA_37°C) | 31.614 | 13.718 | 0.955 |
| Edge one H2O_37°C - (Procodile Q NaOCl+EDTA_37°C) | -65.186 | 13.718 | **0.002** |
| Edge one H2O_37°C - (Reciproc Blue NaOCl+EDTA_37°C) | -49.286 | 13.718 | 0.141 |
| Edge one H2O_37°C - (CC One NaOCl+EDTA_60°C) | 45.814 | 13.718 | 0.269 |
| Edge one H2O_37°C - (Edge one NaOCl+EDTA_60°C) | 73.214 | 13.718 | **<0.001** |
| Edge one H2O_37°C - (Procodile Q NaOCl+EDTA_60°C) | -28.386 | 13.718 | 0.991 |
| Edge one H2O_37°C - (Reciproc Blue NaOCl+EDTA_60°C) | -16.586 | 13.718 | 1.0 |
| Procodile Q H2O_37°C - Reciproc Blue H2O_37°C | 82.111 | 13.122 | **<0.001** |
| Procodile Q H2O_37°C - CC One H2O_60°C | 94.619 | 14.028 | **<0.001** |
| Procodile Q H2O_37°C - Edge one H2O_60°C | 184.708 | 13.526 | **<0.001** |
| Procodile Q H2O_37°C - Procodile Q H2O_60°C | 14.619 | 14.028 | 1.0 |
| Procodile Q H2O_37°C - Reciproc Blue H2O_60°C | 96.333 | 13.122 | **<0.001** |
| Procodile Q H2O_37°C - CC One NaOCl_37°C | 124.458 | 13.526 | **<0.001** |
| Procodile Q H2O_37°C - Edge one NaOCl_37°C | 207.333 | 15.526 | **<0.001** |
| Procodile Q H2O_37°C - Procodile Q NaOCl_37°C | 35.433 | 12.790 | 0.71 |
| Procodile Q H2O_37°C - Reciproc Blue NaOCl_37°C | 99.778 | 13.122 | **<0.001** |
| Procodile Q H2O_37°C - CC One NaOCl_60°C | 214.619 | 14.028 | **<0.001** |
| Procodile Q H2O_37°C - Edge one NaOCl_60°C | 215.833 | 13.526 | **<0.001** |
| Procodile Q H2O_37°C - Procodile Q NaOCl_60°C | 61.833 | 13.526 | **0.004** |
| Procodile Q H2O_37°C - Reciproc Blue NaOCl_60°C | 108.133 | 15.526 | **<0.001** |
| Procodile Q H2O_37°C - (CC One NaOCl+EDTA_37°C) | 184.433 | 12.790 | **<0.001** |
| Procodile Q H2O_37°C - (Edge one NaOCl+EDTA_37°C) | 214.233 | 12.790 | **<0.001** |
| Procodile Q H2O_37°C - (Procodile Q NaOCl+EDTA_37°C) | 117.433 | 12.790 | **<0.001** |
| Procodile Q H2O_37°C - (Reciproc Blue NaOCl+EDTA_37°C) | 133.333 | 12.790 | **<0.001** |
| Procodile Q H2O_37°C - (CC One NaOCl+EDTA_60°C) | 228.433 | 12.790 | **<0.001** |
| Procodile Q H2O_37°C - (Edge one NaOCl+EDTA_60°C) | 255.833 | 12.790 | **<0.001** |
| Procodile Q H2O_37°C - (Procodile Q NaOCl+EDTA_60°C) | 154.233 | 12.790 | **<0.001** |
| Procodile Q H2O_37°C - (Reciproc Blue NaOCl+EDTA_60°C) | 166.033 | 12.790 | **<0.001** |
| Reciproc Blue H2O_37°C - CC One H2O_60°C | 12.508 | 14.028 | 1.0 |
| Reciproc Blue H2O_37°C - Edge one H2O_60°C | 102.597 | 13.526 | **<0.001** |
| Reciproc Blue H2O_37°C - Procodile Q H2O_60°C | -67.492 | 14.028 | **0.002** |
| Reciproc Blue H2O_37°C - Reciproc Blue H2O_60°C | 14.222 | 13.122 | 1.0 |
| Reciproc Blue H2O_37°C - CC One NaOCl_37°C | 42.347 | 13.526 | 0.416 |
| Reciproc Blue H2O_37°C - Edge one NaOCl_37°C | 125.222 | 15.526 | **<0.001** |
| Reciproc Blue H2O_37°C - Procodile Q NaOCl_37°C | -46.678 | 12.790 | 0.12 |
| Reciproc Blue H2O_37°C - Reciproc Blue NaOCl_37°C | 17.667 | 13.122 | 1.0 |
| Reciproc Blue H2O_37°C - CC One NaOCl_60°C | 132.508 | 14.028 | **<0.001** |
| Reciproc Blue H2O_37°C - Edge one NaOCl_60°C | 133.722 | 13.526 | **<0.001** |
| Reciproc Blue H2O_37°C - Procodile Q NaOCl_60°C | -20.278 | 13.526 | 1.0 |
| Reciproc Blue H2O_37°C - Reciproc Blue NaOCl_60°C | 26.022 | 15.526 | 1.0 |
| Reciproc Blue H2O_37°C - (CC One NaOCl+EDTA_37°C) | 102.322 | 12.790 | **<0.001** |
| Reciproc Blue H2O_37°C - (Edge one NaOCl+EDTA_37°C) | 132.122 | 12.790 | **<0.001** |
| Reciproc Blue H2O_37°C - (Procodile Q NaOCl+EDTA_37°C) | 35.322 | 12.790 | 0.717 |
| Reciproc Blue H2O_37°C - (Reciproc Blue NaOCl+EDTA_37°C) | 51.222 | 12.790 | **0.039** |
| Reciproc Blue H2O_37°C - (CC One NaOCl+EDTA_60°C) | 146.322 | 12.790 | **<0.001** |
| Reciproc Blue H2O_37°C - (Edge one NaOCl+EDTA_60°C) | 173.722 | 12.790 | **<0.001** |
| Reciproc Blue H2O_37°C - (Procodile Q NaOCl+EDTA_60°C) | 72.122 | 12.790 | **<0.001** |
| Reciproc Blue H2O_37°C - (Reciproc Blue NaOCl+EDTA_60°C) | 83.922 | 12.790 | **<0.001** |
| CC One H2O_60°C - Edge one H2O_60°C | 90.089 | 14.407 | **<0.001** |
| CC One H2O_60°C - Procodile Q H2O_60°C | -80.000 | 14.879 | **<0.001** |
| CC One H2O_60°C - Reciproc Blue H2O_60°C | 1.714 | 14.028 | 1.0 |
| CC One H2O_60°C - CC One NaOCl_37°C | 29.839 | 14.407 | 0.99 |
| CC One H2O_60°C - Edge one NaOCl_37°C | 112.714 | 16.299 | **<0.001** |
| CC One H2O_60°C - Procodile Q NaOCl_37°C | -59.186 | 13.718 | **0.013** |
| CC One H2O_60°C - Reciproc Blue NaOCl_37°C | 5.159 | 14.028 | 1.0 |
| CC One H2O_60°C - CC One NaOCl_60°C | 120.000 | 14.879 | **<0.001** |
| CC One H2O_60°C - Edge one NaOCl_60°C | 121.214 | 14.407 | **<0.001** |
| CC One H2O_60°C - Procodile Q NaOCl_60°C | -32.786 | 14.407 | 0.962 |
| CC One H2O_60°C - Reciproc Blue NaOCl_60°C | 13.514 | 16.299 | 1.0 |
| CC One H2O_60°C - (CC One NaOCl+EDTA_37°C) | 89.814 | 13.718 | **<0.001** |
| CC One H2O_60°C - (Edge one NaOCl+EDTA_37°C) | 119.614 | 13.718 | **<0.001** |
| CC One H2O_60°C - (Procodile Q NaOCl+EDTA_37°C) | 22.814 | 13.718 | 1.0 |
| CC One H2O_60°C - (Reciproc Blue NaOCl+EDTA_37°C) | 38.714 | 13.718 | 0.669 |
| CC One H2O_60°C - (CC One NaOCl+EDTA_60°C) | 133.814 | 13.718 | **<0.001** |
| CC One H2O_60°C - (Edge one NaOCl+EDTA_60°C) | 161.214 | 13.718 | **<0.001** |
| CC One H2O_60°C - (Procodile Q NaOCl+EDTA_60°C) | 59.614 | 13.718 | **0.011** |
| CC One H2O_60°C - (Reciproc Blue NaOCl+EDTA_60°C) | 71.414 | 13.718 | **<0.001** |
| Edge one H2O_60°C - Procodile Q H2O_60°C | -170.089 | 14.407 | **<0.001** |
| Edge one H2O_60°C - Reciproc Blue H2O_60°C | -88.375 | 13.526 | **<0.001** |
| Edge one H2O_60°C - CC One NaOCl_37°C | -60.250 | 13.918 | **0.012** |
| Edge one H2O_60°C - Edge one NaOCl_37°C | 22.625 | 15.869 | 1.0 |
| Edge one H2O_60°C - Procodile Q NaOCl_37°C | -149.275 | 13.204 | **<0.001** |
| Edge one H2O_60°C - Reciproc Blue NaOCl_37°C | -84.931 | 13.526 | **<0.001** |
| Edge one H2O_60°C - CC One NaOCl_60°C | 29.911 | 14.407 | 0.99 |
| Edge one H2O_60°C - Edge one NaOCl_60°C | 31.125 | 13.918 | 0.97 |
| Edge one H2O_60°C - Procodile Q NaOCl_60°C | -122.875 | 13.918 | **<0.001** |
| Edge one H2O_60°C - Reciproc Blue NaOCl_60°C | -76.575 | 15.869 | **0.002** |
| Edge one H2O_60°C - (CC One NaOCl+EDTA_37°C) | -0.275 | 13.204 | 1.0 |
| Edge one H2O_60°C - (Edge one NaOCl+EDTA_37°C) | 29.525 | 13.204 | 0.97 |
| Edge one H2O_60°C - (Procodile Q NaOCl+EDTA_37°C) | -67.275 | 13.204 | **<0.001** |
| Edge one H2O_60°C - (Reciproc Blue NaOCl+EDTA_37°C) | -51.375 | 13.204 | 0.057 |
| Edge one H2O_60°C - (CC One NaOCl+EDTA_60°C) | 43.725 | 13.204 | 0.286 |
| Edge one H2O_60°C - (Edge one NaOCl+EDTA_60°C) | 71.125 | 13.204 | **<0.001** |
| Edge one H2O_60°C - (Procodile Q NaOCl+EDTA_60°C) | -30.475 | 13.204 | 0.954 |
| Edge one H2O_60°C - (Reciproc Blue NaOCl+EDTA_60°C) | -18.675 | 13.204 | 1.0 |
| Procodile Q H2O_60°C - Reciproc Blue H2O_60°C | 81.714 | 14.028 | **<0.001** |
| Procodile Q H2O_60°C - CC One NaOCl_37°C | 109.839 | 14.407 | **<0.001** |
| Procodile Q H2O_60°C - Edge one NaOCl_37°C | 192.714 | 16.299 | **<0.001** |
| Procodile Q H2O_60°C - Procodile Q NaOCl_37°C | 20.814 | 13.718 | 1.0 |
| Procodile Q H2O_60°C - Reciproc Blue NaOCl_37°C | 85.159 | 14.028 | **<0.001** |
| Procodile Q H2O_60°C - CC One NaOCl_60°C | 200.000 | 14.879 | **<0.001** |
| Procodile Q H2O_60°C - Edge one NaOCl_60°C | 201.214 | 14.407 | **<0.001** |
| Procodile Q H2O_60°C - Procodile Q NaOCl_60°C | 47.214 | 14.407 | 0.309 |
| Procodile Q H2O_60°C - Reciproc Blue NaOCl_60°C | 93.514 | 16.299 | **<0.001** |
| Procodile Q H2O_60°C - (CC One NaOCl+EDTA_37°C) | 169.814 | 13.718 | **<0.001** |
| Procodile Q H2O_60°C - (Edge one NaOCl+EDTA_37°C) | 199.614 | 13.718 | **<0.001** |
| Procodile Q H2O_60°C - (Procodile Q NaOCl+EDTA_37°C) | 102.814 | 13.718 | **<0.001** |
| Procodile Q H2O_60°C - (Reciproc Blue NaOCl+EDTA_37°C) | 118.714 | 13.718 | **<0.001** |
| Procodile Q H2O_60°C - (CC One NaOCl+EDTA_60°C) | 213.814 | 13.718 | **<0.001** |
| Procodile Q H2O_60°C - (Edge one NaOCl+EDTA_60°C) | 241.214 | 13.718 | **<0.001** |
| Procodile Q H2O_60°C - (Procodile Q NaOCl+EDTA_60°C) | 139.614 | 13.718 | **<0.001** |
| Procodile Q H2O_60°C - (Reciproc Blue NaOCl+EDTA_60°C) | 151.414 | 13.718 | **<0.001** |
| Reciproc Blue H2O_60°C - CC One NaOCl_37°C | 28.125 | 13.526 | 0.99 |
| Reciproc Blue H2O_60°C - Edge one NaOCl_37°C | 111.000 | 15.526 | **<0.001** |
| Reciproc Blue H2O_60°C - Procodile Q NaOCl_37°C | -60.900 | 12.790 | **0.002** |
| Reciproc Blue H2O_60°C - Reciproc Blue NaOCl_37°C | 3.444 | 13.122 | 1.0 |
| Reciproc Blue H2O_60°C - CC One NaOCl_60°C | 118.286 | 14.028 | **<0.001** |
| Reciproc Blue H2O_60°C - Edge one NaOCl_60°C | 119.500 | 13.526 | **<0.001** |
| Reciproc Blue H2O_60°C - Procodile Q NaOCl_60°C | -34.500 | 13.526 | 0.857 |
| Reciproc Blue H2O_60°C - Reciproc Blue NaOCl_60°C | 11.800 | 15.526 | 1.0 |
| Reciproc Blue H2O_60°C - (CC One NaOCl+EDTA_37°C) | 88.100 | 12.790 | **<0.001** |
| Reciproc Blue H2O_60°C - (Edge one NaOCl+EDTA_37°C) | 117.900 | 12.790 | **<0.001** |
| Reciproc Blue H2O_60°C - (Procodile Q NaOCl+EDTA_37°C) | 21.100 | 12.790 | 1.0 |
| Reciproc Blue H2O_60°C - (Reciproc Blue NaOCl+EDTA_37°C) | 37.000 | 12.790 | 0.612 |
| Reciproc Blue H2O_60°C - (CC One NaOCl+EDTA_60°C) | 132.100 | 12.790 | **<0.001** |
| Reciproc Blue H2O_60°C - (Edge one NaOCl+EDTA_60°C) | 159.500 | 12.790 | **<0.001** |
| Reciproc Blue H2O_60°C - (Procodile Q NaOCl+EDTA_60°C) | 57.900 | 12.790 | **0.005** |
| Reciproc Blue H2O_60°C - (Reciproc Blue NaOCl+EDTA_60°C) | 69.700 | 12.790 | **<0.001** |
| CC One NaOCl_37°C - Edge one NaOCl_37°C | 82.875 | 15.869 | **<0.001** |
| CC One NaOCl_37°C - Procodile Q NaOCl_37°C | -89.025 | 13.204 | **<0.001** |
| CC One NaOCl_37°C - Reciproc Blue NaOCl_37°C | -24.681 | 13.526 | 0.999 |
| CC One NaOCl_37°C - CC One NaOCl_60°C | 90.161 | 14.407 | **<0.001** |
| CC One NaOCl_37°C - Edge one NaOCl_60°C | 91.375 | 13.918 | **<0.001** |
| CC One NaOCl_37°C - Procodile Q NaOCl_60°C | -62.625 | 13.918 | **0.006** |
| CC One NaOCl_37°C - Reciproc Blue NaOCl_60°C | -16.325 | 15.869 | 1.0 |
| CC One NaOCl_37°C - (CC One NaOCl+EDTA_37°C) | 59.975 | 13.204 | **0.005** |
| CC One NaOCl_37°C - (Edge one NaOCl+EDTA_37°C) | 89.775 | 13.204 | **<0.001** |
| CC One NaOCl_37°C - (Procodile Q NaOCl+EDTA_37°C) | -7.025 | 13.204 | 1.0 |
| CC One NaOCl_37°C - (Reciproc Blue NaOCl+EDTA_37°C) | 8.875 | 13.204 | 1.0 |
| CC One NaOCl_37°C - (CC One NaOCl+EDTA_60°C) | 103.975 | 13.204 | **<0.001** |
| CC One NaOCl_37°C - (Edge one NaOCl+EDTA_60°C) | 131.375 | 13.204 | **<0.001** |
| CC One NaOCl_37°C - (Procodile Q NaOCl+EDTA_60°C) | 29.775 | 13.204 | 0.966 |
| CC One NaOCl_37°C - (Reciproc Blue NaOCl+EDTA_60°C) | 41.575 | 13.204 | 0.402 |
| Edge one NaOCl_37°C - Procodile Q NaOCl_37°C | -171.900 | 15.247 | **<0.001** |
| Edge one NaOCl_37°C - Reciproc Blue NaOCl_37°C | -107.556 | 15.526 | **<0.001** |
| Edge one NaOCl_37°C - CC One NaOCl_60°C | 7.286 | 16.299 | 1.0 |
| Edge one NaOCl_37°C - Edge one NaOCl_60°C | 8.500 | 15.869 | 1.0 |
| Edge one NaOCl_37°C - Procodile Q NaOCl_60°C | -145.500 | 15.869 | **<0.001** |
| Edge one NaOCl_37°C - Reciproc Blue NaOCl_60°C | -99.200 | 17.605 | **<0.001** |
| Edge one NaOCl_37°C - (CC One NaOCl+EDTA_37°C) | -22.900 | 15.247 | 1.0 |
| Edge one NaOCl_37°C - (Edge one NaOCl+EDTA_37°C) | 6.900 | 15.247 | 1.0 |
| Edge one NaOCl_37°C - (Procodile Q NaOCl+EDTA_37°C) | -89.900 | 15.247 | **<0.001** |
| Edge one NaOCl_37°C - (Reciproc Blue NaOCl+EDTA_37°C) | -74.000 | 15.247 | **0.001** |
| Edge one NaOCl_37°C - (CC One NaOCl+EDTA_60°C) | 21.100 | 15.247 | 1.0 |
| Edge one NaOCl_37°C - (Edge one NaOCl+EDTA_60°C) | 48.500 | 15.247 | 0.378 |
| Edge one NaOCl_37°C - (Procodile Q NaOCl+EDTA_60°C) | -53.100 | 15.247 | 0.189 |
| Edge one NaOCl_37°C - (Reciproc Blue NaOCl+EDTA_60°C) | -41.300 | 15.247 | 0.756 |
| Procodile Q NaOCl_37°C - Reciproc Blue NaOCl_37°C | 64.344 | 12.790 | **<0.001** |
| Procodile Q NaOCl_37°C - CC One NaOCl_60°C | 179.186 | 13.718 | **<0.001** |
| Procodile Q NaOCl_37°C - Edge one NaOCl_60°C | 180.400 | 13.204 | **<0.001** |
| Procodile Q NaOCl_37°C - Procodile Q NaOCl_60°C | 26.400 | 13.204 | 0.995 |
| Procodile Q NaOCl_37°C - Reciproc Blue NaOCl_60°C | 72.700 | 15.247 | **0.002** |
| Procodile Q NaOCl_37°C - (CC One NaOCl+EDTA_37°C) | 149.000 | 12.449 | **<0.001** |
| Procodile Q NaOCl_37°C - (Edge one NaOCl+EDTA_37°C) | 178.800 | 12.449 | **<0.001** |
| Procodile Q NaOCl_37°C - (Procodile Q NaOCl+EDTA_37°C) | 82.000 | 12.449 | **<0.001** |
| Procodile Q NaOCl_37°C - (Reciproc Blue NaOCl+EDTA_37°C) | 97.900 | 12.449 | **<0.001** |
| Procodile Q NaOCl_37°C - (CC One NaOCl+EDTA_60°C) | 193.000 | 12.449 | **<0.001** |
| Procodile Q NaOCl_37°C - (Edge one NaOCl+EDTA_60°C) | 220.400 | 12.449 | **<0.001** |
| Procodile Q NaOCl_37°C - (Procodile Q NaOCl+EDTA_60°C) | 118.800 | 12.449 | **<0.001** |
| Procodile Q NaOCl_37°C - (Reciproc Blue NaOCl+EDTA_60°C) | 130.600 | 12.449 | **<0.001** |
| Reciproc Blue NaOCl_37°C - CC One NaOCl_60°C | 114.841 | 14.028 | **<0.001** |
| Reciproc Blue NaOCl_37°C - Edge one NaOCl_60°C | 116.056 | 13.526 | **<0.001** |
| Reciproc Blue NaOCl_37°C - Procodile Q NaOCl_60°C | -37.944 | 13.526 | 0.683 |
| Reciproc Blue NaOCl_37°C - Reciproc Blue NaOCl_60°C | 8.356 | 15.526 | 1.0 |
| Reciproc Blue NaOCl_37°C - (CC One NaOCl+EDTA_37°C) | 84.656 | 12.790 | **<0.001** |
| Reciproc Blue NaOCl_37°C - (Edge one NaOCl+EDTA_37°C) | 114.456 | 12.790 | **<0.001** |
| Reciproc Blue NaOCl_37°C - (Procodile Q NaOCl+EDTA_37°C) | 17.656 | 12.790 | 1.0 |
| Reciproc Blue NaOCl_37°C - (Reciproc Blue NaOCl+EDTA_37°C) | 33.556 | 12.790 | 0.814 |
| Reciproc Blue NaOCl_37°C - (CC One NaOCl+EDTA_60°C) | 128.656 | 12.790 | **<0.001** |
| Reciproc Blue NaOCl_37°C - (Edge one NaOCl+EDTA_60°C) | 156.056 | 12.790 | **<0.001** |
| Reciproc Blue NaOCl_37°C - (Procodile Q NaOCl+EDTA_60°C) | 54.456 | 12.790 | **0.016** |
| Reciproc Blue NaOCl_37°C - (Reciproc Blue NaOCl+EDTA_60°C) | 66.256 | 12.790 | **<0.001** |
| CC One NaOCl_60°C - Edge one NaOCl_60°C | 1.214 | 14.407 | 1.0 |
| CC One NaOCl_60°C - Procodile Q NaOCl_60°C | -152.786 | 14.407 | **<0.001** |
| CC One NaOCl_60°C - Reciproc Blue NaOCl_60°C | -106.486 | 16.299 | **<0.001** |
| CC One NaOCl_60°C - (CC One NaOCl+EDTA_37°C) | -30.186 | 13.718 | 0.976 |
| CC One NaOCl_60°C - (Edge one NaOCl+EDTA_37°C) | -0.386 | 13.718 | 1.0 |
| CC One NaOCl_60°C - (Procodile Q NaOCl+EDTA_37°C) | -97.186 | 13.718 | **<0.001** |
| CC One NaOCl_60°C - (Reciproc Blue NaOCl+EDTA_37°C) | -81.286 | 13.718 | **<0.001** |
| CC One NaOCl_60°C - (CC One NaOCl+EDTA_60°C) | 13.814 | 13.718 | 1.0 |
| CC One NaOCl_60°C - (Edge one NaOCl+EDTA_60°C) | 41.214 | 13.718 | 0.519 |
| CC One NaOCl_60°C - (Procodile Q NaOCl+EDTA_60°C) | -60.386 | 13.718 | **0.009** |
| CC One NaOCl_60°C - (Reciproc Blue NaOCl+EDTA_60°C) | -48.586 | 13.718 | 0.162 |
| Edge one NaOCl_60°C - Procodile Q NaOCl_60°C | -154.000 | 13.918 | **<0.001** |
| Edge one NaOCl_60°C - Reciproc Blue NaOCl_60°C | -107.700 | 15.869 | **<0.001** |
| Edge one NaOCl_60°C - (CC One NaOCl+EDTA_37°C) | -31.400 | 13.204 | 0.933 |
| Edge one NaOCl_60°C - (Edge one NaOCl+EDTA_37°C) | -1.600 | 13.204 | 1.0 |
| Edge one NaOCl_60°C - (Procodile Q NaOCl+EDTA_37°C) | -98.400 | 13.204 | **<0.001** |
| Edge one NaOCl_60°C - (Reciproc Blue NaOCl+EDTA_37°C) | -82.500 | 13.204 | **<0.001** |
| Edge one NaOCl_60°C - (CC One NaOCl+EDTA_60°C) | 12.600 | 13.204 | 1.0 |
| Edge one NaOCl_60°C - (Edge one NaOCl+EDTA_60°C) | 40.000 | 13.204 | 0.498 |
| Edge one NaOCl_60°C - (Procodile Q NaOCl+EDTA_60°C) | -61.600 | 13.204 | **0.003** |
| Edge one NaOCl_60°C - (Reciproc Blue NaOCl+EDTA_60°C) | -49.800 | 13.204 | 0.083 |
| Procodile Q NaOCl_60°C - Reciproc Blue NaOCl_60°C | 46.300 | 15.869 | 0.591 |
| Procodile Q NaOCl_60°C - (CC One NaOCl+EDTA_37°C) | 122.600 | 13.204 | **<0.001** |
| Procodile Q NaOCl_60°C - (Edge one NaOCl+EDTA_37°C) | 152.400 | 13.204 | **<0.001** |
| Procodile Q NaOCl_60°C - (Procodile Q NaOCl+EDTA_37°C) | 55.600 | 13.204 | **0.019** |
| Procodile Q NaOCl_60°C - (Reciproc Blue NaOCl+EDTA_37°C) | 71.500 | 13.204 | **<0.001** |
| Procodile Q NaOCl_60°C - (CC One NaOCl+EDTA_60°C) | 166.600 | 13.204 | **<0.001** |
| Procodile Q NaOCl_60°C - (Edge one NaOCl+EDTA_60°C) | 194.000 | 13.204 | **<0.001** |
| Procodile Q NaOCl_60°C - (Procodile Q NaOCl+EDTA_60°C) | 92.400 | 13.204 | **<0.001** |
| Procodile Q NaOCl_60°C - (Reciproc Blue NaOCl+EDTA_60°C) | 104.200 | 13.204 | **<0.001** |
| Reciproc Blue NaOCl_60°C - (CC One NaOCl+EDTA_37°C) | 76.300 | 15.247 | **<0.001** |
| Reciproc Blue NaOCl_60°C - (Edge one NaOCl+EDTA_37°C) | 106.100 | 15.247 | **<0.001** |
| Reciproc Blue NaOCl_60°C - (Procodile Q NaOCl+EDTA_37°C) | 9.300 | 15.247 | 1.0 |
| Reciproc Blue NaOCl_60°C - (Reciproc Blue NaOCl+EDTA_37°C) | 25.200 | 15.247 | 1.0 |
| Reciproc Blue NaOCl_60°C - (CC One NaOCl+EDTA_60°C) | 120.300 | 15.247 | **<0.001** |
| Reciproc Blue NaOCl_60°C - (Edge one NaOCl+EDTA_60°C) | 147.700 | 15.247 | **<0.001** |
| Reciproc Blue NaOCl_60°C - (Procodile Q NaOCl+EDTA_60°C) | 46.100 | 15.247 | 0.503 |
| Reciproc Blue NaOCl_60°C - (Reciproc Blue NaOCl+EDTA_60°C) | 57.900 | 15.247 | 0.077 |
| (CC One NaOCl+EDTA_37°C) - (Edge one NaOCl+EDTA_37°C) | 29.800 | 12.449 | 0.928 |
| (CC One NaOCl+EDTA_37°C) - (Procodile Q NaOCl+EDTA_37°C) | -67.000 | 12.449 | **<0.001** |
| (CC One NaOCl+EDTA_37°C) - (Reciproc Blue NaOCl+EDTA_37°C) | -51.100 | 12.449 | **0.027** |
| (CC One NaOCl+EDTA_37°C) - (CC One NaOCl+EDTA_60°C) | 44.000 | 12.449 | 0.165 |
| (CC One NaOCl+EDTA_37°C) - (Edge one NaOCl+EDTA_60°C) | 71.400 | 12.449 | **<0.001** |
| (CC One NaOCl+EDTA_37°C) - (Procodile Q NaOCl+EDTA_60°C) | -30.200 | 12.449 | 0.916 |
| (CC One NaOCl+EDTA_37°C) - (Reciproc Blue NaOCl+EDTA_60°C) | -18.400 | 12.449 | 1.0 |
| (Edge one NaOCl+EDTA_37°C) - (Procodile Q NaOCl+EDTA_37°C) | -96.800 | 12.449 | **<0.001** |
| (Edge one NaOCl+EDTA_37°C) - (Reciproc Blue NaOCl+EDTA_37°C) | -80.900 | 12.449 | **<0.001** |
| (Edge one NaOCl+EDTA_37°C) - (CC One NaOCl+EDTA_60°C) | 14.200 | 12.449 | 1.0 |
| (Edge one NaOCl+EDTA_37°C) - (Edge one NaOCl+EDTA_60°C) | 41.600 | 12.449 | 0.267 |
| (Edge one NaOCl+EDTA_37°C) - (Procodile Q NaOCl+EDTA_60°C) | -60.000 | 12.449 | **0.002** |
| (Edge one NaOCl+EDTA_37°C) - (Reciproc Blue NaOCl+EDTA_60°C) | -48.200 | 12.449 | 0.061 |
| (Procodile Q NaOCl+EDTA_37°C) - (Reciproc Blue NaOCl+EDTA_37°C) | 15.900 | 12.449 | 1.0 |
| (Procodile Q NaOCl+EDTA_37°C) - (CC One NaOCl+EDTA_60°C) | 111.000 | 12.449 | **<0.001** |
| (Procodile Q NaOCl+EDTA_37°C) - (Edge one NaOCl+EDTA_60°C) | 138.400 | 12.449 | **<0.001** |
| (Procodile Q NaOCl+EDTA_37°C) - (Procodile Q NaOCl+EDTA_60°C) | 36.800 | 12.449 | 0.559 |
| (Procodile Q NaOCl+EDTA_37°C) - (Reciproc Blue NaOCl+EDTA_60°C) | 48.600 | 12.449 | 0.055 |
| (Reciproc Blue NaOCl+EDTA_37°C) - (CC One NaOCl+EDTA_60°C) | 95.100 | 12.449 | **<0.001** |
| (Reciproc Blue NaOCl+EDTA_37°C) - (Edge one NaOCl+EDTA_60°C) | 122.500 | 12.449 | **<0.001** |
| (Reciproc Blue NaOCl+EDTA_37°C) - (Procodile Q NaOCl+EDTA_60°C) | 20.900 | 12.449 | 1.0 |
| (Reciproc Blue NaOCl+EDTA_37°C) - (Reciproc Blue NaOCl+EDTA_60°C) | 32.700 | 12.449 | 0.812 |
| (CC One NaOCl+EDTA_60°C) - (Edge one NaOCl+EDTA_60°C) | 27.400 | 12.449 | 0.976 |
| (CC One NaOCl+EDTA_60°C) - (Procodile Q NaOCl+EDTA_60°C) | -74.200 | 12.449 | **<0.001** |
| (CC One NaOCl+EDTA_60°C) - (Reciproc Blue NaOCl+EDTA_60°C) | -62.400 | 12.449 | **<0.001** |
| (Edge one NaOCl+EDTA_60°C) - (Procodile Q NaOCl+EDTA_60°C) | -101.600 | 12.449 | **<0.001** |
| (Edge one NaOCl+EDTA_60°C) - (Reciproc Blue NaOCl+EDTA_60°C) | -89.800 | 12.449 | **<0.001** |
| (Procodile Q NaOCl+EDTA_60°C) - (Reciproc Blue NaOCl+EDTA_60°C) | 11.800 | 12.449 | 1.0 |

Significant P values are shown in bold (*P < 0.05).* Abbreviations: EDTA, ethylenediaminetetraacetic acid; NaOCl, sodium hypochlorite SE, standard error; TTF, time to fracture
